# Fission yeast Caprin protein is required for efficient heterochromatin establishment

**DOI:** 10.1371/journal.pgen.1011620

**Published:** 2025-03-10

**Authors:** Haidao Zhang, Ekaterina Kapitonova, Adriana Orrego, Christos Spanos, Joanna Strachan, Elizabeth H. Bayne

**Affiliations:** 1 Institute of Cell Biology, School of Biological Sciences, University of Edinburgh, Edinburgh, United Kingdom; 2 Institute of Molecular Plant Sciences, School of Biological Sciences, University of Edinburgh, Edinburgh, United Kingdom; Justus Liebig Universitat Giessen, GERMANY

## Abstract

Heterochromatin is a key feature of eukaryotic genomes that serves important regulatory and structural roles in regions such as centromeres. In fission yeast, maintenance of existing heterochromatic domains relies on positive feedback loops involving histone methylation and non-coding RNAs. However, requirements for *de novo* establishment of heterochromatin are less well understood. Here, through a cross-based assay we have identified a novel factor influencing the efficiency of heterochromatin establishment. We determine that the previously uncharacterised protein is an ortholog of human Caprin1, an RNA-binding protein linked to stress granule formation. We confirm that the fission yeast ortholog, here named Cpn1, also associates with stress granules, and we uncover evidence of interplay between heterochromatin integrity and ribonucleoprotein (RNP) granule formation, with heterochromatin mutants showing reduced granule formation in the presence of stress, but increased granule formation in the absence of stress. We link this to regulation of non-coding heterochromatic transcripts, since in heterochromatin-deficient cells, Cpn1 can be seen to colocalise with accumulating pericentromeric transcripts, and absence of Cpn1 leads to hyperaccumulation of these RNAs at centromeres. Together, our findings unveil a novel link between RNP homeostasis and heterochromatin assembly, and implicate Cpn1 and associated factors in facilitating efficient heterochromatin establishment by enabling removal of excess transcripts that would otherwise impair assembly processes.

## Author summary

Regulation and stability of eukaryotic genomes is dependent on the assembly of certain regions into a distinct, repressive form of chromatin termed heterochromatin. How pre-existing heterochromatin is propagated is well understood, but much less is known about the early steps of heterochromatin establishment. Here, through a novel genetic screening strategy we identified a new factor required for efficient heterochromatin establishment in fission yeast that we name Cpn1. Cpn1 is the yeast counterpart of human protein CAPRIN1, an RNA binding protein with a role in stress responses. We show here that Cpn1 has similar stress-related functions, and in addition has a role in regulating heterochromatic transcripts. In particular, Cpn1 influences the localisation and degradation of these transcripts, preventing their hyperaccumulation on chromatin in a way that impairs heterochromatin assembly. Our findings highlight clearance of excess transcripts as a key step in enabling efficient heterochromatin establishment. In addition, they have potential implications for the study of human disease: altered expression of human CAPRIN1 is associated with a number of diseases including cancer, but molecular functions of Caprin proteins remain poorly understood; our discovery of Cpn1 in yeast opens new opportunities to study CAPRIN protein family function in a simple model organism.

## Introduction

Heterochromatin is a distinct, condensed form of chromatin that serves important regulatory and structural roles in eukaryotic genomes. It can be divided into two types: facultative heterochromatin contributes to dynamic regulation of gene expression in response to developmental or environmental cues, whereas constitutive heterochromatin is typically found in gene-poor, repeat-rich regions of the genome such as centromeres, where it plays important roles in the maintenance of genome stability including repressing invasive genetic elements and supporting accurate chromosome segregation [1]. Heterochromatin is characterised by relatively low levels of histone acetylation, and in most eukaryotes by enrichment for methylation of histone H3 at lysine 9 (H3K9me). This histone methyl mark provides binding sites for chromodomain proteins including heterochromatin protein HP1, which contributes to chromatin compaction and promotes recruitment of further effector proteins involved in transcriptional silencing, chromatin organisation and chromosome segregation [2].

Paradoxically, although heterochromatin is generally associated with transcriptional repression, local transcription is commonly required to support heterochromatin assembly, and RNA processing and surveillance pathways have been shown to play dual roles in co-/post-transcriptional silencing and RNA-based targeting of chromatin modifiers [2–4]. In the fission yeast *Schizosaccharomyces pombe*, the RNA interference (RNAi) pathway plays a major role in maintenance of constitutive pericentromeric heterochromatin. Non-coding RNAs derived from pericentromeric repeat sequences are processed by Dicer (Dcr1) to generate short interfering (si)RNAs [5–7]. These siRNAs are bound by Argonaute (Ago1) and serve as sequence-specific guides to target the Ago1-containing RITS complex to complementary nascent RNAs [8,9]. Via the adaptor protein Stc1 [10,11], RNA-bound RITS mediates recruitment of the Clr4 complex (CLRC) comprising the H3K9 methyltransferase Clr4 along with several other factors including WD repeat protein Rik1, all of which are required for histone methylation [10,12–15]. The resulting H3K9 methylation in turn helps to stabilise recruitment of both RNAi and heterochromatin factors via binding of chromodomain-containing proteins including the RITS subunit Chp1, HP1 protein Swi6, and Clr4 [16], such that siRNAs and H3K9 methylation together form a self-reinforcing signal loop for heterochromatin maintenance.

In fission yeast, additional RNA-dependent but RNAi-independent pathways have also been found to contribute to heterochromatin assembly, particularly at domains of facultative heterochromatin. These domains include so-called heterochromatin islands, typically associated with meiotic genes that are repressed during vegetative growth [17,18], as well as additional heterochromatin domains (HOODs) that are detectable at retrotransposons and some developmentally regulated genes in certain conditions, including when the nuclear exosome is inactivated [19]. While HOODs are partially RNAi-dependent, heterochromatin islands are RNAi-independent, and both rely to varying degrees on other RNA processing factors, including the exosome adapter Mtl1-Red1 core complex MTREC [20,21], the 5′-3′ exonuclease Dhp1/Xrn2 [22,23], and the Ccr4-Not deadenylase complex [24–27], for coupled RNA elimination and heterochromatin assembly. Absence of the Ccr4-Not complex has been shown to be associated with increased accumulation of heterochromatic transcripts on chromatin that leads to impaired silencing [24], indicating that while RNA production is required to provide a platform for recruitment of heterochromatin assembly factors, excess accumulation of such transcripts can negatively impact heterochromatin integrity.

A key feature of heterochromatin is its capacity for epigenetic inheritance through reader-writer coupling [28]. In fission yeast, high densities of H3K9me3 promote binding of Clr4 and methylation of neighbouring nucleosomes, facilitating propagation of pre-existing heterochromatin [29]. This epigenetic maintenance phase can be distinguished from *de novo* heterochromatin establishment, which occurs through two consecutive stochastic steps: initiation at nucleation sites, followed by subsequent spreading to form extended domains [30]. Dependent on context, some factors and processes required for *de novo* heterochromatin establishment are redundant for subsequent maintenance, necessitating dedicated strategies for their detection. For example, histone deacetylase (HDAC) activity is required to promote heterochromatin assembly by increasing availability of deacetylated H3K9 residues for methylation as well as suppressing acetylation-dependent histone turnover [31]. Deletion of HDACs Sir2 or Clr3 has only modest effects on maintenance of pericentromeric heterochromatin, but strongly impacts *de novo* heterochromatin nucleation and spreading on pericentromeric repeat sequences [32,33]. A small number of additional factors have also been identified as being specifically required for heterochromatin establishment with little or no role in subsequent maintenance, including Triman (Tri1), a 3′-5′ exonuclease involved in siRNA biogenesis [34], and the RNA-binding protein Mkt1 that is involved in RNAi-dependent post-transcriptional regulation of heterochromatic transcripts [35]. We previously showed that Mkt1, similar to Ccr4-Not complex, is required to prevent excess accumulation of transcripts on chromatin and formation of RNA-DNA hybrids, suggesting that excess RNA may be a particular impediment to the initial conversion of highly transcribed chromatin to heterochromatin.

Here, through an assay involving genetic abolition and re-establishment of heterochromatin, we identify the product of a previously uncharacterised gene, SPAC12G12.07c, as a novel factor required for efficient heterochromatin establishment in *S. pombe*. The SPAC12G12.07c gene product is orthologous to human RNA-binding protein CAPRIN1, and we therefore name it Cpn1. Human CAPRIN1 is involved in stress granule formation, and we confirm that fission yeast Cpn1 both associates with stress granule-related factors, and accumulates in granules during stress. Moreover, we find evidence of interplay between heterochromatin integrity and RNP granule formation, suggesting the involvement of common limiting factors in control of both transcripts released from polysomes during stress, and those accumulating as a result of de-repression of heterochromatin. Consistent with this, RNA-FISH analyses revealed that, when heterochromatin is disrupted, Cpn1 co-localises with non-coding pericentromeric transcripts, and absence of Cpn1 is associated with hyperaccumulation of these transcripts that localise *in cis* at centromeres. Together, our findings unveil a role for Cpn1 in supporting efficient heterochromatin establishment by enabling the removal of excess heterochromatic transcripts.

## Results

### Efficiency of heterochromatin establishment is influenced by parental genetic background

**Fig 1:**
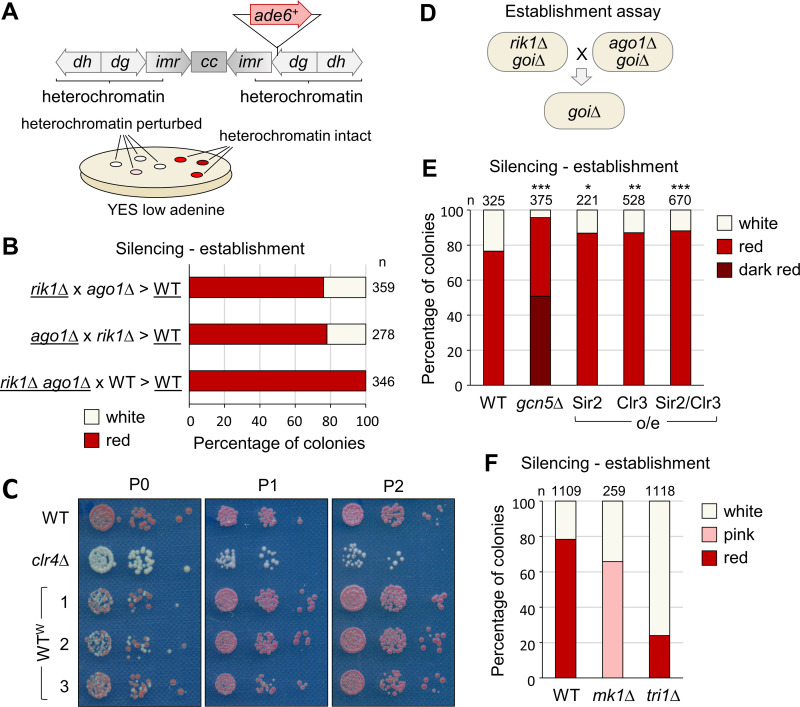
Efficiency of heterochromatin re-establishment is influenced by genetic background. (A) Schematic representation of the *cen1:ade6*^*+*^ reporter system. The position of the *ade6*^*+*^ insertion at centromere one is shown relative to the centromeric outer repeats (*dg* and *dh*), innermost repeats (*imr*) and central core (*cc*). Intact heterochromatin is associated with silencing of *cen1:ade6*^*+*^ reporter resulting in red colonies on low adenine media; loss of heterochromatin alleviates silencing giving pink/white colonies. (B) Proportions of red (*cen1:ade6*^*+*^ silenced) versus white (*cen1:ade6*^*+*^ expressed) colonies in the wild-type progeny of crosses between parental strains of the indicated genotypes (underlining denotes strains carrying the *cen1:ade6*^*+*^ reporter), based on analysis of *n* colonies. (C) Passaging of wild-type ‘white’ (WT^W^) colonies selected from B. (D) Schematic of the cross-based establishment assay. (E) and (F) Proportions of red (*cen1:ade6*^*+*^ silenced) versus pink/white (*cen1:ade6*^*+*^ expressed) colonies in the otherwise wild-type progeny of *rik1∆* x *ago1∆* crosses performed in the indicated genetic backgrounds, based on analysis of *n* colonies. Relative to wild-type, asterisks denote p ≤ 0.05 (*), p ≤ 0.01 (**) or p ≤ 0.001 (***) from chi-squared (χ^2^) test analysis.

To investigate factors influencing heterochromatin establishment, we conducted crosses with different CLRC and/or RNAi mutant strains deficient in heterochromatin, and examined the efficiency of heterochromatin re-establishment in the wild-type progeny. An *ade6*^*+*^ reporter gene inserted into the heterochromatic outer repeats of centromere one (*cen1:ade6*^*+*^) was employed as a readout: in cells lacking centromeric heterochromatin this reporter is expressed resulting in pale/white colonies, whereas assembly of heterochromatin causes silencing of the reporter resulting in red colonies (Fig 1A). When a reporter-carrying strain lacking heterochromatin (e.g., *rik1∆ ago1∆* double mutant, defective for both H3K9 methylation and RNAi), was crossed to a wild-type strain without the reporter, the resulting wild-type (*rik1+ ago1+*), reporter-carrying progeny were 100% red, consistent with the expected re-establishment of heterochromatin and silencing in this background (Fig 1B). Interestingly, however, when we crossed together two different heterochromatin-deficient strains, e.g., *rik1∆* and *ago1∆* single mutants, we observed a mix of red and white colonies in the wild-type progeny (~80% red, 20% white). Similar results were seen when crossing other pairs of heterochromatin-deficient strains, although the ratios of red:white colonies varied with parental genotype (S1A Fig). In contrast, the ratio of red:white colonies was highly consistent amongst crosses of different, independent *ago1∆* and *rik1∆* strains (S1B Fig). It was also consistent regardless of the parent of origin of the reporter (Fig 1B). Passaging of progeny colonies revealed that while the red, silenced state is stable, the white phenotype tends to switch to red over time, consistent with slow heterochromatin re-establishment (Fig 1C). Together these results indicate that, upon reintroduction of missing heterochromatin-related factors by crossing, the efficiency of heterochromatin establishment is dependent on the specific parental background.

We next tested whether the rate of heterochromatin re-establishment in the progeny resulting from a cross between *ago1∆* and *rik1∆* strains could be modulated by backgrounds expected to promote or suppress heterochromatin assembly. Since histone deacetylation is required to enable stable H3K9 methylation, we reasoned that the rate of heterochromatin establishment might be increased by interventions that reduce histone H3 acetylation. To test this, we repeated the cross in backgrounds either lacking the histone acetyltransferase Gcn5 [36,37], or over-expressing deacetylases Sir2 and/or Clr3. For example, we deleted *gcn5*^*+*^ in *ago1∆* and *rik1∆* strains, crossed the resulting strains together, and analysed the *gcn5∆ ago1+ rik1+* progeny (Fig 1D). As predicted, we found that removal of Gcn5 was sufficient to increase the efficiency of heterochromatin establishment, resulting in the proportion of red colonies in the cross progeny increasing to almost 100% (Fig 1E). Over-expression of Sir2 and/or Clr3 also resulted in a smaller but still significant increase in red colony proportion, consistent with their reported roles in promoting heterochromatin establishment [32,33]. Conversely, we also tested the impact of deleting two factors previously shown to promote heterochromatin establishment, Mkt1 and Tri1 [34,35]. As expected, in the absence of either of these factors the efficiency of heterochromatin establishment appeared reduced, with *tri1∆* progeny showing a strong reduction in the proportion of red colonies, and *mkt1∆* progeny showing a mixture of white and pink but no red colonies (Fig 1F). Thus, this cross-based system can serve as a sensitized assay to detect and quantify changes in efficiency of heterochromatin establishment.

### Cpn1 is a novel factor required for efficient heterochromatin establishment

**Fig 2:**
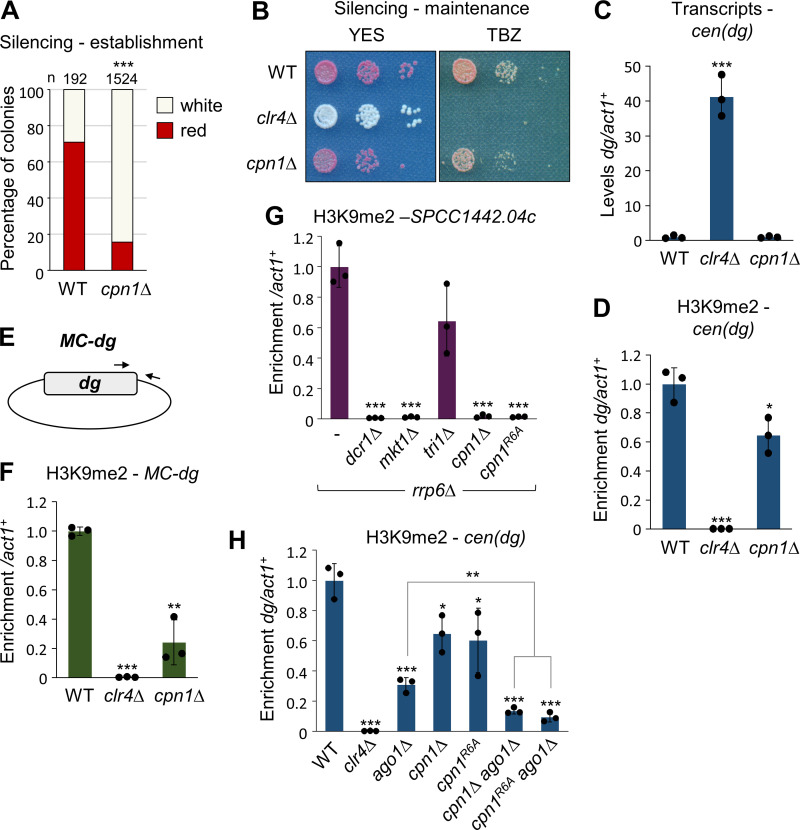
Cpn1 is a novel factor required for efficient heterochromatin establishment. (A) Assay for establishment of silencing of the *cen1:ade6*^*+*^ reporter. Shown are proportions of red (*ade6*^*+*^ silenced) versus white (*ade6*^*+*^ expressed) colonies in the otherwise wild-type progeny of *rik1∆* x *ago1∆* cross performed in wild-type or *cpn1∆* background (as in 1D), based on analysis of *n* colonies. Asterisks denote p ≤ 0.001 (***) from chi-squared (χ^2^) test analysis. (B) Assay for maintenance of silencing of the *cen1:ade6*^*+*^ reporter (red colonies indicate silencing; white colonies loss of silencing), and for sensitivity to TBZ (20 μg/μl). (C) RT-qPCR analysis of *cen*(*dg*) transcript levels relative to *act1*^*+*^, normalised to wild-type. (D) ChIP-qPCR analysis of H3K9me2 levels at *cen*(*dg*) relative to *act1*^*+*^, normalised to wild-type. (E) Schematic of the minichromosome (*MC-dg*) plasmid. (F) ChIP-qPCR analysis of H3K9me2 levels established *de novo* on the *MC-dg* plasmid, relative to *act1*^*+*^, normalised to wild-type. (G) ChIP-qPCR analysis of H3K9me2 levels at HOOD locus *SPCC1442.04c,* relative to *act1*^*+*^, normalised to wild-type. (H) ChIP-qPCR analysis of H3K9me2 levels at *cen*(*dg*) relative to *act1*^*+*^, normalised to wild-type. In each case data are averages of three biological replicates; dots represent individual data points and error bars represent one SD. Asterisks denote p ≤ 0.05 (*), p ≤ 0.01 (**) or p ≤ 0.001 (***) from Student’s t-test analysis.

As a starting point to uncover potential novel heterochromatin establishment factors, we returned to data from a previous genetic screen for factors influencing heterochromatin maintenance [38]. In that screen, maintenance of silencing of the *cen1:ade6*^*+*^ reporter was primarily assessed via colony growth on media lacking adenine, which was scored semi-quantitatively on a scale of one to four, where four indicated expression comparable to wild-type, and one indicated strong de-repression. 75 mutant strains were given a growth score of three, suggesting possible mild de-repression of the reporter, but falling below the threshold (of two) set for further follow-up analysis as a candidate maintenance factor. We reasoned that these strains showing potential slight defects in heterochromatin maintenance might include deletions of factors that play a greater role in heterochromatin establishment. To further narrow down the list we selected factors with no known *S. cerevisiae* ortholog (according to PomBase [39]), since H3K9 methylation and RNAi are absent in this species. This resulted in a shortlist of 13 genes, one of which was known establishment factor Tri1, validating the approach. Of the remaining 12 genes, three were previously uncharacterised, and of these one in particular stood out: SPAC12G12.07c, hereon named *cpn1*^*+*^ for reasons outlined below. Like another known establishment factor, Mkt1, the product of this gene has previously been reported to physically associate with components of the MTREC exosome adaptor complex that is implicated in maintenance of facultative heterochromatin domains [40], making it a strong candidate as a novel pathway component. To test this, we assessed the impact of *cpn1*^*+*^ deletion in our *rik1∆* x *ago1∆* establishment assay. Strikingly, *cpn1∆* progeny showed a clear reduction in the proportion of red colonies, similar to what was seen in the *tri1∆* background, suggesting that the product of this gene is required for efficient heterochromatin establishment (Fig 2A).

Our previous screen results had suggested that deletion of *cpn1*^*+*^ might result in a small defect in heterochromatin maintenance [38]. To assess this in more detail, we deleted *cpn1*^*+*^ in a wild-type strain carrying the *cen1:ade6*^*+*^ reporter to assess the effect on heterochromatin maintenance. Colony colour of *cpn1∆* strains was indistinguishable from wild-type, suggesting little or no effect on maintenance of reporter gene silencing (Fig 2B). Consistent with this, *cpn1∆* strains, in contrast to *clr4∆* strains, showed little increase in sensitivity to the microtubule destabilising drug TBZ, indicating that centromere function is maintained. This was further supported by RT-qPCR analysis that showed no significant increase in accumulation of non-coding pericentromeric (*dg*) transcripts (Fig 2C). In addition, ChIP-qPCR analysis of H3K9me2 revealed only a small reduction in H3K9 methylation levels at pericentromeric repeat sequences (Fig 2D). Hence loss of Cpn1 function has little effect on heterochromatin maintenance, and rather appears to particularly affect heterochromatin establishment.

As a complementary approach to further verify and characterise the heterochromatin establishment defects in *cpn1∆* cells, we employed an alternative assay whereby we assessed *de novo* heterochromatin establishment on a newly introduced minichromosome, as described previously [32,35]. Wild-type and mutant strains were transformed with a minichromosome plasmid (*MC-dg*) that carries a 5.6kb portion of centromeric (*dg*) outer-repeat sequence that is targeted by endogenous centromeric siRNAs (Fig 2E). ChIP-qPCR analysis revealed that, whereas in wild-type cells, heterochromatin is efficiently established on the plasmid resulting in high levels of H3K9me2, in cells lacking Cpn1 the levels of H3K9me2 established on the plasmid are substantially reduced (Fig 2F). This is consistent with the reduced efficiency of re-initiation of pericentromeric reporter gene silencing observed in *cpn1∆* cells, and supports the conclusion that absence of Cpn1 is associated with defective heterochromatin establishment.

Cpn1 has previously been reported to interact with MTREC components including Red1, Red5, Mtl1 and Pir2 [40]. We therefore tested whether Cpn1 also functions together with MTREC in facilitating assembly of facultative heterochromatin. Two classes of MTREC-dependent facultative heterochromatin domains can be distinguished: heterochromatin islands, which are RNAi-independent and typically serve to silence meiotic genes during vegetative growth [17,18], and so-called HOODs, which are dependent on both MTREC and RNAi and form at loci such as transposons in the absence of Rrp6 [19]. In the case of heterochromatin islands, RT-qPCR analysis revealed that, while absence of MTREC component Red1 results in loss of silencing of meiotic genes such as *mei4*^*+*^ and *ssm4*^*+*^, the removal of Cpn1 has no effect at these loci (S2A Fig). Similarly, at HOODs (assessed in *rrp6∆* background), ChIP-qPCR analysis indicated that, whereas H3K9 methylation is lost upon deletion of *dcr1*^*+*^, it is retained at most loci tested in the absence of *cpn1*^*+*^ (S2B Fig). However, the one exception to this is the HOOD associated with meiotic gene SPCC1442.04c, where we found heterochromatin to be strongly dependent on functional Cpn1 (Fig 2G). This was striking since we previously found that this particular HOOD (but not other HOODs or islands) is also dependent on Mkt1 (but not Tri1) [35]. This HOOD is also unusual in being Red1-independent [19]. Hence Cpn1, like Mkt1, plays a role in supporting RNAi-dependent facultative heterochromatin formation in certain contexts, possibly acting in parallel to Red1.

The commonalities described above prompted us to probe whether Mkt1 and Cpn1 might be functionally linked. We showed previously that Mkt1 acts in the same pathway as RNAi for pericentromeric silencing. To test if this is also true for Cpn1, we assessed pericentromeric H3K9me2 levels in *cpn1∆ ago1∆* double mutant cells. In contrast to our previous observations for Mkt1, we found that the *cpn1∆ ago1∆* double mutants displayed additive defects in H3K9 methylation as compared to either single mutant, suggesting independent effects (Fig 2H). Hence it appears that while Mkt1 functions together with RNAi, Cpn1 functions in another, separate pathway.

### Cpn1 is a CAPRIN family protein

**Fig 3:**
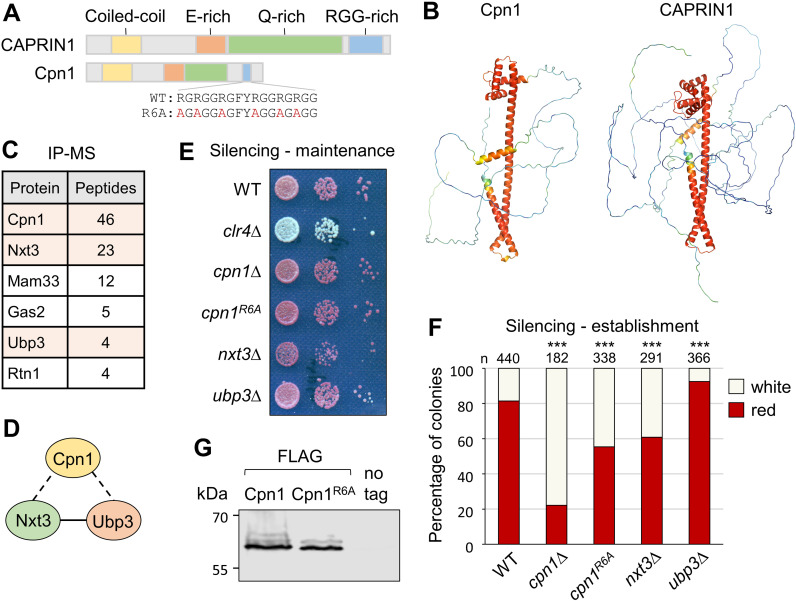
Cpn1 is a CAPRIN family protein. (A) Schematic representation of CAPRIN proteins in human (CAPRIN1) and *S. pombe* (Cpn1). (B) Alphafold structural models of Cpn1 and CAPRIN1. (C) List of proteins found specifically and reproducibly associated with Cpn1-FLAG by affinity purification and mass spectrometry (LC-MS/MS). Shown are total peptide counts for all proteins represented by ≥ 2 peptides in purifications from Cpn1-FLAG strains, but not untagged control strains, in two independent experiments. (D) Putative Cpn1 complex (dotted lines denote interactions detected here; solid line indicates interaction detected previously by IP-western [45]). (E) Assay for maintenance of silencing of *cen1:ade6*^*+*^ reporter: red colonies on low adenine media indicate silencing, and white colonies absence of silencing. (F) Assay for establishment of silencing of *cen1:ade6*^*+*^ reporter: shown are proportions of red (*ade6*^*+*^ silenced) versus white (*ade6*^*+*^ expressed) colonies in the otherwise wild-type progeny of *rik1∆* x *ago1∆* crosses performed in the indicated deletion backgrounds (as in 1D), based on analysis of *n* colonies. Relative to wild-type, asterisks denote p ≤ 0.001 (***) from chi-squared (χ^2^) test analysis. (G) Western blot analysis of affinity-purified FLAG-tagged wild-type Cpn1 and Cpn1^R6A^.

At the time of this study no orthologs of the SPAC12G12.07c gene product had been identified outside of fungi. However, through an iterative search for remote homology using JACKHMMER [41], we identified 1:1 orthology with human protein CAPRIN1. Although they have only 15% identity at the amino level, the human and fission yeast proteins share similar motif organisation, including N-terminal coiled-coil and C-terminal RG/RGG-rich regions (Fig 3A). Moreover, comparison of structural models generated by AlphaFold [42,43] also reveals clear similarities in structural organisation (Fig 3B). We therefore named the SPAC12G12.07c gene product as fission yeast Caprin protein, Cpn1.

Human CAPRIN1 is a ubiquitously expressed mRNA-binding protein that is known to be involved in regulation of stress granule formation in association with two partner proteins, G3BP1 and USP10 [44]. To investigate fission yeast Cpn1 interaction partners, we C-terminally FLAG-tagged Cpn1 at the endogenous locus and performed immunoprecipitation followed by liquid chromatography-tandem mass spectrometry (LC-MS/MS) to identify associated proteins. Only five proteins were specifically and reproducibly identified in precipitates from Cpn1-FLAG-expressing cells but not controls, and strikingly, two of these were Nxt3 and Ubp3, the fission yeast orthologs of human CAPRIN1 partner proteins G3BP1 and USP10, respectively (Fig 3C and 3D). Nxt3 and Ubp3 are themselves known to interact, and to localise to stress granules [45,46]. The association with Nxt3 and Ubp3 further supports the conclusion that fission yeast Cpn1 is the functional ortholog of human CAPRIN1.

To explore whether Nxt3 and Ubp3 also function together with Cpn1 to influence heterochromatin assembly, we generated *nxt3∆* and *ubp3∆* deletion strains and tested them in heterochromatin maintenance and establishment assays. While absence of these partner proteins, like loss of Cpn1 itself, had little effect on heterochromatin maintenance (Fig 3E), it did affect heterochromatin establishment in our *rik1∆* x *ago1∆*. Interestingly, the two deletions had opposing effects: while in *nxt3∆* cells, as in *cpn1∆* cells, the efficiency of heterochromatin establishment was reduced (lower proportion of red colonies in the cross progeny compared to wild-type), in *ubp3∆* cells we conversely saw a small increase in establishment efficiency (increased proportion of red colonies; Fig 3F). Considering what is known about their human orthologs, this may be explained by opposing functions of these proteins, since CAPRIN1 and G3BP1 are both RNA-binding proteins that have been shown act cooperatively to promote stress granule assembly, while USP10, which lacks an RNA-binding domain, acts to limit assembly [44]. Hence Cpn1 acts together with its interaction partners to influence heterochromatin establishment efficiency.

The RGG-rich region in human CAPRIN1 has been shown to be important for RNA-binding and stress granule formation [47]. To assess whether the RG/RGG-rich motif in fission yeast Cpn1 is also required for its function in heterochromatin establishment, we generated strains in which each of the six arginine residues in this region were mutated to alanine (Cpn1^R6A^; Fig 3A). Testing this mutant in our *rik1∆* x *ago1∆* establishment assay, we observed a reduction in the proportion of red colonies in the progeny compared to wild-type, indicating reduced efficiency of heterochromatin establishment, although the effect was not as strong as that associated with *cpn1*^*+*^ deletion (Fig 3F). Moreover, in Cpn1^R6A^-expressing cells we also observed loss of H3K9me2 from the SPCC1442.04c HOOD locus, and a reduction in H3K9me2 maintenance at pericentromeric repeats in *ago1∆* background, comparable to that in *cpn1∆* cells (Fig 2G and 2H). Western blot analysis of FLAG-tagged wild-type and mutant Cpn1 proteins showed no difference in protein accumulation (Fig 3G), confirming that the mutations do not affect protein stability. Hence the RGG-rich region contributes to Cpn1 function in facilitating heterochromatin assembly.

Since the RGG motifs in human CAPRIN1 are involved in RNA binding, and since we have shown previously that Mkt1 interacts with non-coding pericentromeric transcripts [35], we tested whether Cpn1 might also bind to pericentromeric RNAs. As previously, we performed RNA-IPs in cells lacking Sir2, to relieve transcriptional repression at pericentromeric heterochromatin [35]. We detected specific enrichment of pericentromeric transcripts (*dg* and *imr*) in Mkt1-FLAG pulldowns relative to controls, consistent with our previous results. However, no such enrichment was observed in Cpn1-FLAG pull-downs, suggesting that Cpn1 does not stably bind these RNAs (S3 Fig). We also tested whether association of Mkt1 with pericentromeric transcripts is dependent on Cpn1, but again found no evidence of this, with both *dg* and *imr* transcripts being similarly enriched in Mkt1-FLAG pull downs in the presence and absence Cpn1 (S3 Fig). This supports the conclusion that Cpn1 and Mkt1 function independently in heterochromatic silencing.

To explore possible wider effects of Cpn1 on the transcriptome, and rule-out the possibility that loss of Cpn1 might affect heterochromatin establishment indirectly via altered expression of one or more heterochromatin pathway components, we performed transcriptome analysis by RNA sequencing in *cpn1∆* cells. Very few transcripts were found to be differentially expressed in *cpn1∆* cells compared to wildtype (11 up-regulated, 15 down-regulated; S1 and S2 Tables, respectively). Importantly, no genes with known links to heterochromatin assembly showed altered expression, arguing against a possible indirect effect of Cpn1 removal. Interestingly, the majority of upregulated transcripts were non-coding RNAs, including pericentromeric transcripts, as well as transcripts from a small number of other, non-heterochromatic loci, consistent with a role for Cpn1 in non-coding RNA regulation. Moreover, nearly all of the down-regulated transcripts derived from subtelomeric loci (within 100kb of the ends of chromosomes I or II). Given that Cpn1 impacts centromeric heterochromatin assembly, and that subtelomeres are also heterochromatic, we hypothesised that this down-regulation might reflect increased heterochromatinisation in these regions. Consistent with this, ChIP-qPCR analyses revealed a trend towards elevated levels of H3K9 methylation at subtelomeric loci in *cpn1∆* cells (S4 Fig). Increased H3K9 methylation was most clearly detected at sites just outside the normal heterochromatin domain boundaries seen in wild-type cells [48], indicating heterochromatin spreading. Previous studies have indicated that subtelomeric heterochromatin domains have flexible boundaries and can serve as sinks for excess heterochromatin factors [49]; we therefore suspect that redistribution of silencing factors indirectly accounts for the increased repression of subtelomeric genes in *cpn1∆* cells, although we also cannot rule out a more direct role for Cpn1 in regulation at subtelomeres.

### Cpn1 promotes stress granule formation

**Fig 4:**
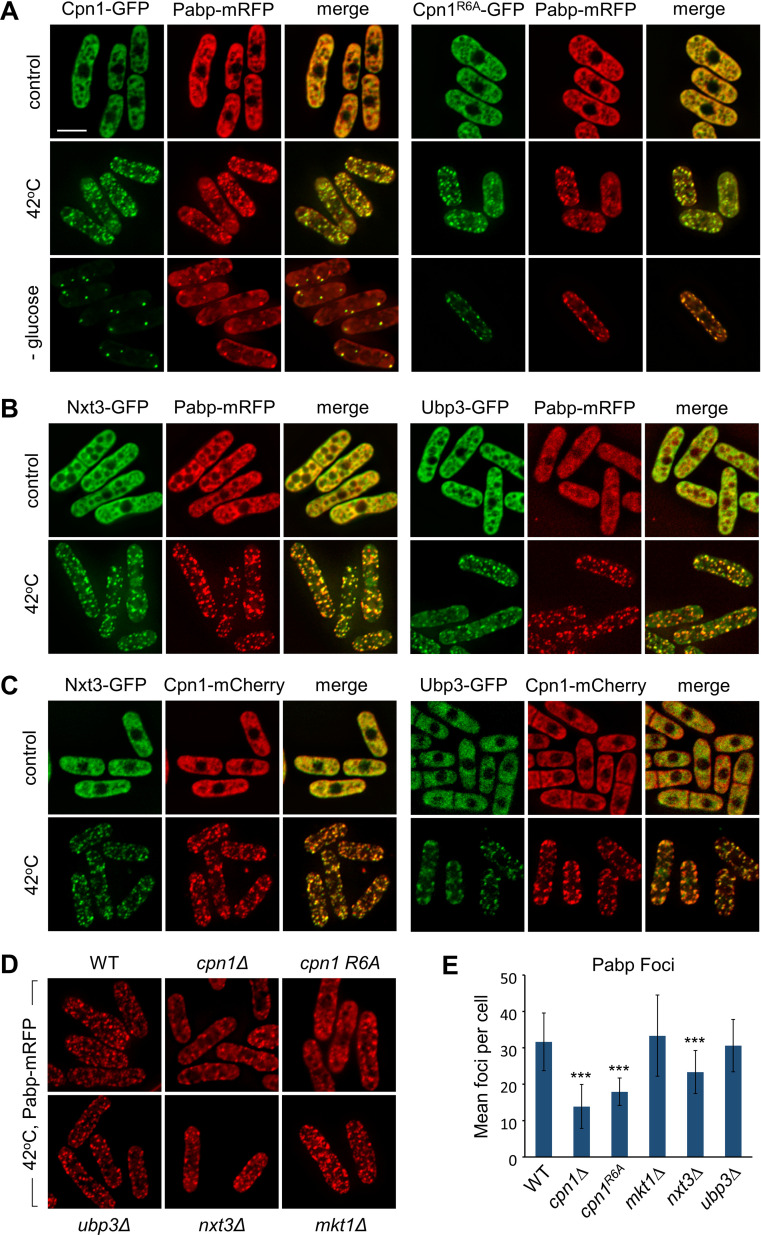
Cpn1 localises to stress granules and promotes their formation. (A) Representative images from two-colour live-cell imaging of Cpn1-GFP or Cpn1^R6A^-GFP (green), and Pabp-mRFP (red), in cells grown at 32^o^C (control) and exposed to 42^o^C heat shock for 20 min, or glucose starvation for 20 min. Bar indicates 6 µm. (B) and (C) Representative images from two-colour live-cell imaging of Nxt3-GFP or Ubp3-GFP (green), and either Pabp-mRFP (red; B) or Cpn1-mCherry (red; C), in cells either untreated or treated with heat shock as above. (D) Representative images from live-cell imaging of Pabp-mRFP (red) in the indicated genetic backgrounds in cells exposed to heat shock as above. (E) Quantification of the mean number of Pabp-mRFP foci per cell from the imaging analysis shown in D (*n* = 100). Error bars represent one SD and asterisks denote p ≤ 0.001 (***) from Student’s t-test analysis.

To test whether Cpn1, like its human ortholog, localises to stress granules, we introduced a C-terminal GFP tag at the endogenous locus and performed live cell imaging analyses. While in unstressed cells we saw a diffuse pattern of localisation, in cells subjected to stress (heat shock or glucose starvation), Cpn1 could be seen to accumulate in discrete cytoplasmic foci. Analysis of cells co-expressing stress granule marker poly(A)-binding protein Pabp tagged with RFP [50] indicated that Cpn1 colocalises with Pabp, confirming that these foci correspond to stress granules (Fig 4A). Similar results were obtained for GFP-tagged Cpn1^R6A^, indicating that the RGG-rich region is not required for Cpn1 localisation to stress granules. We also generated strains expressing C-terminally GFP-tagged Nxt3 or Ubp3, and confirmed that both of these proteins also colocalise with both Pabp and Cpn1 in stress-induced granules (Fig 4B and 4C). Western blot and heterochromatin establishment assays confirmed that all of the GFP-tagged proteins are stably expressed and functional (S5 Fig). Hence Cpn1, together with its partner proteins Nxt3 and Ubp3, does indeed partition into phase-separated, membrane-less organelles in response to stress.

Human CAPRIN1 has also been shown to be required to promote stress granule assembly, in a manner dependent on its RGG repeats [44,47]. To test if the same is true for fission yeast Cpn1, we quantified Pabp focus formation in response to stress in cells lacking Cpn1 or other factors. In line with predictions, in cells lacking Cpn1 we found that the average number of Pabp foci formed per cell following heat shock was substantially reduced (Fig 4D and 4E). Granule formation was also reduced in cells expressing Cpn1^R6A^, suggesting that the RGG-rich motif is important for Cpn1 function in stress granule assembly. We also observed a reduction in granules in cells lacking Nxt3, consistent with findings for the human ortholog G3BP1 [44]. In contrast, no change in Pabp focus formation was observed in cells lacking Ubp3, or Mkt1 (Fig 4D and 4E). Hence like their human orthologs, Cpn1 and Nxt3 are required to promote stress granule formation.

### Interplay between heterochromatin integrity and cytoplasmic RNP granule formation

**Fig 5:**
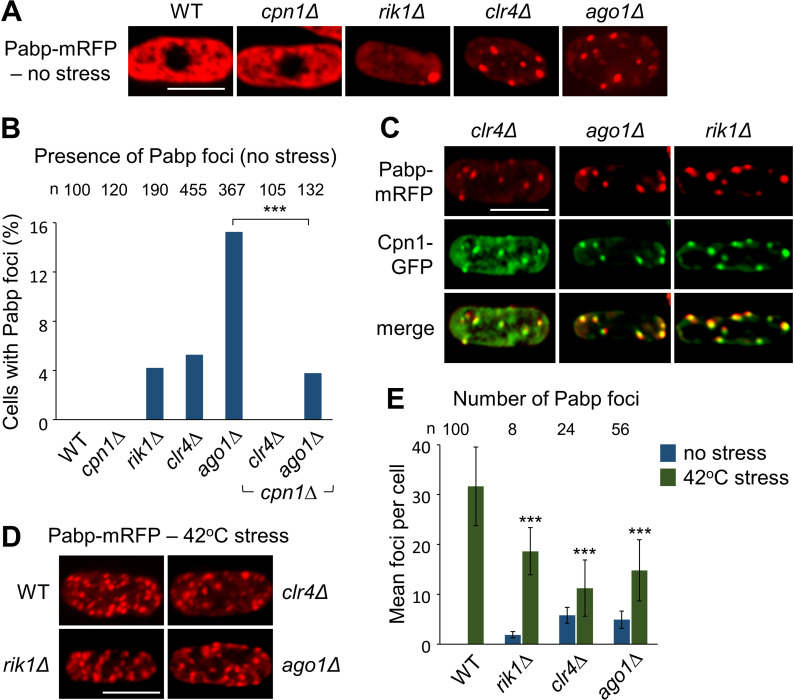
Disruption of heterochromatin is associated with altered formation of Pabp-containing RNP granules. (A) Representative images from live-cell imaging of Pabp-mRFP (red) in the indicated genetic backgrounds in unstressed cells. Bar indicates 6 µm. (B) Quantification of the proportion of unstressed cells displaying Pabp-mRFP foci from the imaging analysis shown in A, based on analysis of *n* cells. Asterisks denote p ≤ 0.001 (***) from chi-squared (χ^2^) test analysis. (C) Representative images from two-colour live-cell imaging of Cpn1-GFP (green), and Pabp-mRFP (red), in the indicated genetic backgrounds in unstressed cells. (D) Representative images from live-cell imaging of Pabp-mRFP (red) in the indicated genetic backgrounds in cells exposed to 42^o^C heat shock for 20 min. (E) Quantification of the mean number of Pabp-mRFP foci per cell in the indicated genetic backgrounds in unstressed cells (based on analysis of *n* cells containing foci from the imaging analysis shown in A), or cells exposed to 42^o^C heat shock for 20 min (*n* = 100 from the imaging analysis shown in D; virtually all cells contained foci). Error bars represent one SD and asterisks denote p ≤ 0.001 (***) from Student’s t-test analysis.

We next set out to explore the possible connection between assembly of heterochromatin and RNP granules. First, we tested whether mutations that disrupt heterochromatin impact on granule formation. Interestingly, whereas in the wild-type background, Pabp foci are not seen in unstressed cells, when we deleted genes encoding components of either the RNAi machinery (Ago1) or the CLRC H3K9 methyltransferase complex (Clr4 or Rik1), we observed a proportion of cells exhibiting Pabp foci even in the absence of stress (Fig 5A and 5B). This was most prominent in *ago1∆* cells, where granules were visible in approximately 15% of cells. Analysis of cells co-expressing Cpn1-GFP confirmed that Cpn1 also localises to these granules, as for canonical stress granules (Fig 5C; colocalization was seen in all cells displaying visible granules). We further tested whether the formation of these *ago1∆* or *clr4∆*-dependent granules is dependent on Cpn1. Indeed, we found that granule formation was greatly reduced in *cpn1∆ ago1∆* or *cpn1∆ clr4∆* double mutants compared to the respective single mutants, indicating that Cpn1 is required to promote the formation of these heterochromatin deficiency-induced granules (Fig 5B). Western blot analysis indicated that Cpn1 protein levels are unaffected by absence of Clr4, Rik1, or Ago1, confirming that the appearance of granules in heterochromatin mutants is not due to altered Cpn1 expression (S6 Fig). Similarly, previous expression analyses have shown that loss of Clr4 or Ago1 has little overall effect on the transcriptome in standard conditions [51–54], arguing against the possibility that increased granule formation reflects a global change in mRNA abundance.

We also tested what happens in heterochromatin-deficient cells in the presence of stress. Interestingly, we found that, whereas heterochromatin mutants show increased Pabp granule formation in absence of stress, they show a significant reduction in the average number of Pabp foci formed in response to heat shock, compared to wild-type cells (Fig 5D). Analysis of foci area confirmed that mean focus size was unchanged in the mutants, but mean foci area per cell reduced, indicating reduced stress granule formation in these backgrounds (S7A Fig). Surprisingly, the same effect was not seen in cells subject to glucose starvation, suggesting differences in the responses to these two forms of stress (S7B and S7C Fig). Together, these observations suggest a degree of interplay between heterochromatin integrity and stress granule formation that could reflect involvement of one or more common limiting factors.

### Cpn1 colocalises with heterochromatic transcripts

**Fig 6:**
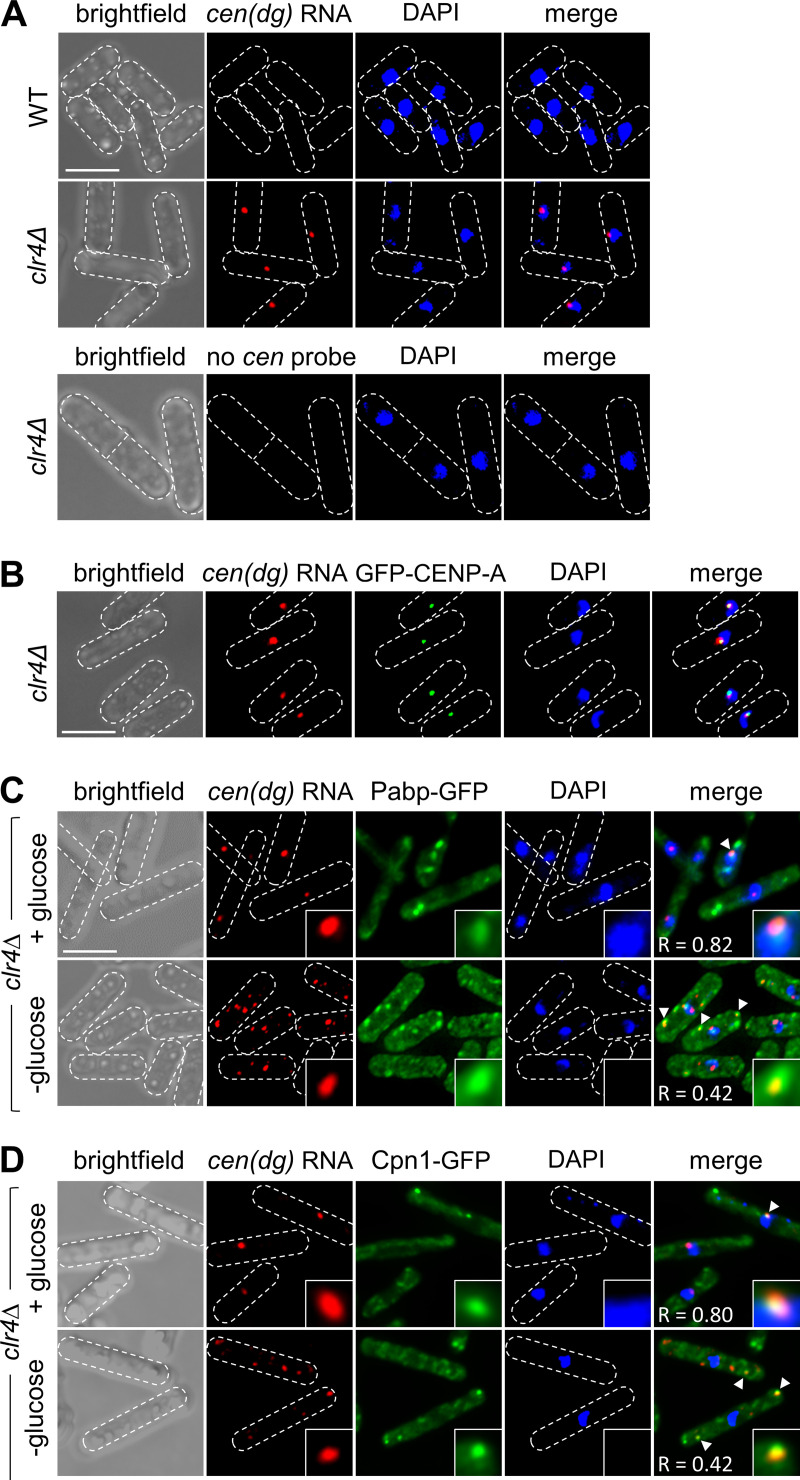
Cpn1 colcalises with heterochromatic transcripts in heterochromatin deficient cells. (A) Representative images from smRNA-FISH analysis of pericentromeric *(cen(dg))* RNA in wild-type or *clr4∆* strains. Data are representative of three independent experiments. Bar indicates 6 µm. (B) Representative images from simultaneous analysis of *cen(dg)* RNA by smRNA-FISH, and GFP-CENP-A^Cnp1^, in *clr4∆* cells. Data are representative of two independent experiments. (C) and (D) Representative images from simultaneous analysis of *cen(dg)* RNA by smRNA-FISH, and Pabp-GFP (C), or Cpn1-GFP (D), in *clr4∆* cells either unstressed (+ glucose) or stressed by 20 min of glucose starvation (- glucose). Arrow heads highlight examples of co-localisation (Pearson’s correlation coefficient, *R*, is indicated, and inserts show 5x magnification). Data are representative of at least three independent experiments.

We hypothesised that the RNP granules that are occasionally formed in response to heterochromatin disruption may be linked to sequestration and/or degradation of excess heterochromatic transcripts that accumulate in the absence of heterochromatin-associated silencing. While previous fractionation analyses have provided evidence that heterochromatic transcripts tend to be retained on chromatin [24], to our knowledge there has been no direct visualisation of heterochromatic transcript subcellular localisation in *S. pombe*. To explore whether the RNP granules formed in response to heterochromatin disruption might harbour non-coding pericentromeric RNAs, we therefore set out determine the subcellular localisation of these RNAs by single‐molecule mRNA fluorescence *in situ* hybridization (smRNA-FISH) using probes targeting *cen*(*dg*) transcripts. Previous RT-qPCR and northern blot analyses have indicated that pericentromeric transcript levels are low in wild-type cells, but greatly elevated in heterochromatin mutants where transcriptional silencing is alleviated [6,10,55,56]. Consistent with this, by smRNA-FISH we could detect a strong signal for *cen*(*dg*) transcripts in *clr4∆* cells, but not in wild-type cells, confirming that the signal is cen(*dg*) transcript-specific (Fig 6A). Notably, *cen*(*dg*) transcripts were detected in one strong focus in the nucleus, which we suspected might corresponded to the position of centromeres, which tend to cluster together at the nuclear periphery in *S. pombe* [57]. To test this, we combined smRNA-FISH with analysis of GFP-tagged centromeric histone variant CENP-A^Cnp1^. The *cen*(*dg*) transcript focus was consistently found adjacent to, or overlapping, the CENP-A^Cnp1^ focus, consistent with transcripts being primarily retained at the pericentromeres (Fig 6B).

To address whether *cen*(*dg*) transcripts might also localise to the Pabp foci seen in a small proportion of (unstressed) heterochromatin-deficient cells, we combined RNA-FISH with simultaneous analysis of Pabp-GFP. In those *clr4∆* cells where visible Pabp foci were formed, we detected no additional *cen*(*dg*) transcript foci, and hence no co-localisation of these transcripts with Pabp foci in the cytoplasm (Fig 6C, upper panel). This suggests that cytoplasmic Pabp foci formed in this background are not sites of stable heterochromatic transcript accumulation. However, in almost half of *clr4∆* cells with Pabp foci, a nuclear Pabp focus could be observed, and this nuclear Pabp signal frequently co-localised with the *cen*(*dg*) transcript focus (Pearson’s correlation coefficient, R_p_ = 0.82, for the 43% of cells displaying nuclear Pabp foci). Nuclear colocalisation of Pabp with *cen*(*dg*) transcripts was also observed in a proportion of *ago1∆* cells (S8A Fig, upper panel; R_p_ = 0.85, for the 24% of cells displaying nuclear Pabp foci). Moreover, visualisation of Cpn1-GFP in *clr4∆* cells similarly revealed some localisation of Cpn1 in the nucleus (in 35% of cells displaying Cpn1-GFP foci), with the nuclear Cpn1-GFP focus also often colocalising with *cen*(*dg*) transcripts (Fig 6D upper panel; R_p_ = 0.80). Hence, disruption of heterochromatin is associated with some accumulation of the predominantly cytoplasmic Cpn1 protein in the nucleus; this observation is consistent with a previous study that showed accumulation of Cpn1 in the nucleus following treatment of cells with leptomycin B, which inhibits CRM1-dependent nuclear export [58]. That Cpn1 can enter the nucleus and co-localise with accumulating heterochromatic transcripts is consistent with it playing a direct role in their regulation.

Although the cytoplasmic Pabp/Cpn1 foci occasionally seen in unstressed, heterochromatin-deficient cells appeared not to be sites of stable *cen*(*dg*) transcript accumulation, it remained possible that heterochromatic transcripts might be exported to the cytoplasm, possibly for degradation. Moreover, co-localisation of Cpn1 and Pabp with heterochromatic transcripts in the nucleus of unstressed cells suggested that these transcripts might be expected to show altered localisation in conditions of stress, where Pabp and Cpn1 consistently relocalise into cytoplasmic granules. To explore these possibilities, we performed additional RNA-FISH analyses in *clr4∆* or *ago1∆* cells subject to stress. Notably, following glucose starvation, additional *cen*(*dg*) transcript foci were observed in the cytoplasm, at least some of which colocalised with Pabp-GFP-marked stress granules (Figs 6C and S8A, lower panels, R_p_ = 0.42 and 0.40, respectively). The stress-induced cytoplasmic *cen*(*dg*) transcript foci also showed co-localisation with Cpn1-GFP (Fig 6D, lower panel, R_p_ = 0.42). No DAPI signal was detected in association with these cytoplasmic *cen*(*dg*) transcript foci, arguing against the possibility that they arise as a result of micronuclei formation. Hence these observations indicate that heterochromatic transcripts can exit the nucleus, and co-localise with Cpn1 and associated factors in the cytoplasm when these proteins relocalise during stress.

### Cpn1 helps limit accumulation of heterochromatic transcripts on chromatin

**Fig 7:**
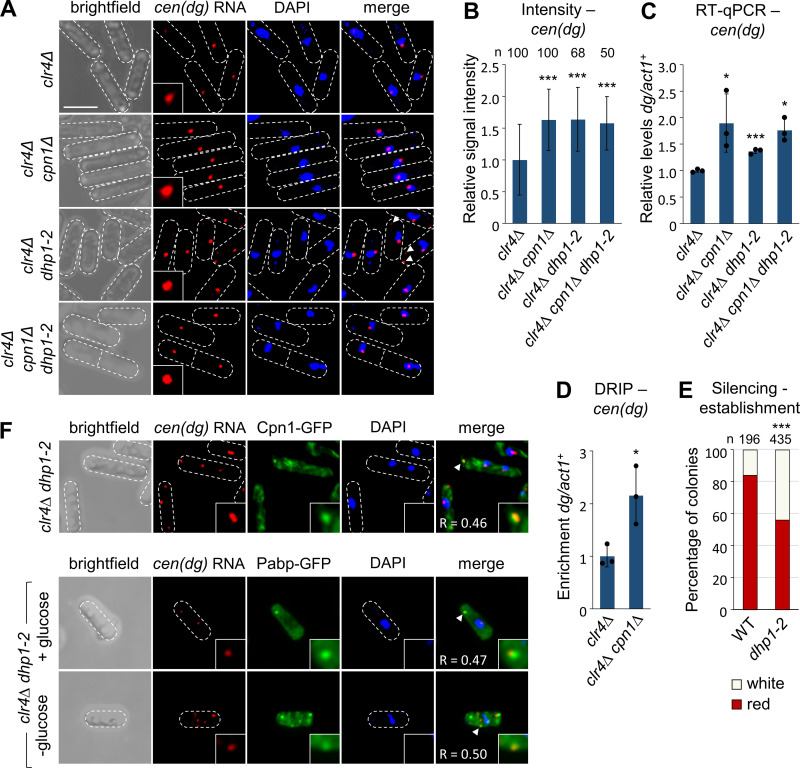
Absence of Cpn1 results in increased accumulation of heterochromatic transcripts at centromeres in heterochromatin deficient cells. (A) Representative images from smRNA-FISH analysis of *cen(dg)* RNA in *clr4∆*, *clr4∆ cpn1∆*, *clr4∆ dhp1-2*, and *clr4∆ cpn1∆ dhp1-2* cells. Arrow heads highlight examples of cytoplasmic RNA foci; Bar indicates 6 µm. Data are representative of at least three independent experiments. (B) Quantification of the mean signal intensity for nuclear *cen(dg)* RNA foci, normalised to *clr4∆* cells, from the smRNA-FISH analysis shown in A. (C) RT-qPCR analysis of total cellular levels of *cen(dg)* RNA relative to *act1*^*+*^, normalised to *clr4∆* cells. (D) DRIP analysis of DNA:RNA hybrid levels at *cen*(*dg*) relative to *act1*^*+*^, normalised to *clr4∆* cells. Data are averages of three biological replicates, dots represent individual data points, and error bars represent one SD. Asterisks denote p ≤ 0.05 (*), or p ≤ 0.001 (***), from Student’s t-test analysis. (E) Proportions of red (*cen1:ade6*^*+*^ silenced) versus white (*cen1:ade6*^*+*^ expressed) colonies in the otherwise wild-type progeny of *rik1∆* x *ago1∆* crosses performed in the indicated genetic backgrounds, based on analysis of *n* colonies. Asterisks denote p ≤ 0.001 (***) from chi-squared (χ^2^) test analysis. (F) Representative images from simultaneous analysis of *cen(dg)* RNA by smRNA-FISH, and either Pabp-GFP or Cpn1-GFP, in *clr4∆ dhp1-2* cells. Arrow heads highlight examples of co-localisation (Pearson’s correlation coefficient, *R*, is indicated, and inserts show 5x magnification). Data are representative of two independent experiments.

To assess whether Cpn1 impacts pericentromeric transcript localisation and/or accumulation in the absence of stress, we performed further RNA-FISH analyses in cells lacking Cpn1. Interestingly, we observed a visible increase in the intensity of the *cen(dg)* transcript signal at centromeres in *clr4∆ cpn1∆* double mutants compared to *clr4∆* single mutants, suggesting that Cpn1 is required to help limit accumulation of these transcripts at centromeres (Fig 7A and 7B). The same effect was also seen in *ago1∆* background (S8B and S8C Fig). DNA-RNA immunoprecipitation (DRIP) analyses also revealed an increase in DNA-RNA hybrid accumulation at centromeres in *clr4∆ cpn1∆* double mutants compared to *clr4∆* single mutants, consistent with excess RNAs accumulating on chromatin (Fig 7D). In principle, the Cpn1-dependent protection of centromeres from excess RNA accumulation might be achieved either via sequestration of these RNAs elsewhere in the cell, or through their degradation. To distinguish between these two possibilities, we analysed total cellular levels of *cen(dg)* transcripts by RT-qPCR. Strikingly, this revealed that deletion of *cpn1*^*+*^ in either *clr4∆* or *ago1∆* background results in a significant increase in total *cen(dg)* transcripts levels, beyond the already high levels present in absence of Clr4 or Ago1 alone (Figs 7C and S8D). Thus, Cpn1 appears to be required to facilitate degradation of excess heterochromatic transcripts, limiting their accumulation on chromatin.

Finally, we reasoned that if Cpn1 facilitates RNA degradation in the cytoplasm, suppression of degradation (independently of other Cpn1 functions) might lead to visible cytoplasmic accumulation of *cen(dg)* transcripts. Since Caprin proteins do not themselves appear to have nuclease activity, it is likely that Cpn1 primarily influences transcript localisation and/or accessibility for degradation by one or more ribonucleases. We identified the 5’-3’ exoribonuclease Dhp1 (ortholog of human Xrn2) as a likely candidate, since: (1) in human embryonic stem cells, Xrn2 has been implicated in mediating CAPRIN1-dependent degradation of selected transcripts in the cytoplasm [59]; and (2) Dhp1, like Cpn1, has been shown to be required for efficient heterochromatin establishment in *S. pombe* [22, 23]. We confirmed that Dhp1 is required for heterochromatin establishment in our system: because *dhp1*^*+*^ is an essential gene, we tested the effects of a previously-described temperature-sensitive allele, *dhp1-2*, that causes defects in heterochromatin, but little impairment of growth, at the permissive temperature [23]. Performing our *rik1∆* x *ago1∆* establishment assay in the *dhp1-2* mutant background resulted in a reduced proportion of red colonies in the progeny compared to wild-type, similar to what we saw for *cpn1*^*+*^ deletion, consistent with the possibility that these factors might act together in heterochromatin establishment (Fig 7E). To explore the influence of Dhp1 on pericentromeric transcript accumulation, we performed further RNA-FISH analyses in *dhp1-2* mutant cells. Strikingly, whereas in *clr4∆* cells a single *cen(dg)* transcript focus can be seen in the nucleus, in *dhp1-2 clr4∆* cells, we observed the appearance of additional *cen(dg)* RNA foci in the cytoplasm (Fig 7A; cytoplasmic RNA foci were detected in ~22% of *dhp1-2 clr4∆* cells, possibly reflecting the fact that *dhp1-2* is a hypomorphic allele). This indicates that active degradation prevents accumulation of *cen(dg)* transcripts in the cytoplasm in *clr4∆* cells in normal conditions. Importantly, the cytoplasmic *cen(dg)* RNA foci were abolished in *cpn1∆ dhp1-2 clr4∆* triple mutant cells, consistent with loss of Cpn1 causing a defect upstream of Dhp1, likely in the localisation of transcripts for degradation (Fig 7A). Moreover, we confirmed that a proportion of the cytoplasmic *cen(dg)* RNA foci that appear in *dhp1-2 clr4∆* cells show colocalisation with Pabp-GFP, and with Cpn1-GFP (Fig 7F; Pearson’s Correlation Coefficients 0.47 and 0.46, respectively), further supporting their functional association. Together, our data suggest that Cpn1 helps to enable heterochromatic transcript degradation by Dhp1, limiting excess accumulation of RNA on chromatin that may otherwise interfere with heterochromatin assembly.

## Discussion

Multiple RNA processing pathways have been found to contribute to RNA-directed heterochromatin assembly and silencing of associated transcripts. Here we have identified a novel role for the fission yeast ortholog of human CAPRIN1, an RNA-binding protein, in facilitating efficient heterochromatin establishment. Absence of Cpn1 has little effect on the maintenance of pre-existing heterochromatin. However, in cells where heterochromatin is abolished and hence non-coding heterochromatic transcripts accumulate, loss of Cpn1 is associated with build-up of even higher levels heterochromatic RNAs, which we show accumulate *in cis* at centromeres. Previous studies by ourselves and others have provided evidence that accumulation of transcripts on chromatin can impair heterochromatin assembly, possibly through increased formation of RNA-DNA hybrids [24,35], and we show here that loss of Cpn1 is associated with increased RNA-DNA hybrid formation at centromeres. Therefore, together, our data suggest that Cpn1 influences the efficiency of heterochromatin assembly by enabling the degradation of heterochromatic transcripts to prevent their detrimental excessive accumulation on chromatin.

Our study revealed the previously uncharacterised protein SPAC12G12.07c as the fission yeast ortholog of human CAPRIN1. Caprin proteins are well conserved in vertebrates, and have also been identified in some other metazoan groups including insects [60]; this work confirms that they are also present in fungi. Human CAPRIN1 is implicated in regulating the transport, translation and/or degradation of mRNAs linked to various processes including the innate immune response [61] cell proliferation [47,62] and differentiation [59]. However, its best characterised role is in stress granule formation, acting together with partner proteins G3BP1 and USP10. We have confirmed that fission yeast Cpn1 also localises to stress granules, and associates with Nxt3 and Ubp3, which are the orthologs of G3BP1 and USP10, respectively. We also show that Cpn1 is required for granule formation in response to heat shock. This is consistent with studies in human cells that have shown RNA-binding protein G3BP1 to be a key nucleator of stress granules, with CAPRIN1 association acting cooperatively to enhance multivalent interactions and hence promote granule formation, and binding of USP10 (which lacks an RNA-binding domain) conversely ‘capping’ interactions and suppressing granule formation [44,63–65]. In line with this, we show here that absence of G3BP1 ortholog Nxt3, but not Usp10 ortholog Ubp3, impairs for granule formation. Superficially, this contrasts with the conclusion from a previous study that Nxt3 is dispensable for granule formation in fission yeast [45]. However, the analysis in that study was not quantitative, and we show here that granule formation is reduced but not abolished in *nxt3∆* cells, indicating that Nxt3, like Cpn1, enhances, but is not absolutely required for, granule formation in fission yeast.

What could be the relationship between stress granule-associated factors and heterochromatin? We have found evidence of interplay between heterochromatin integrity and formation of cytoplasmic RNP granules, since in heterochromatin mutants we observed both increased Pabp-containing granule formation in the absence of stress, and decreased granule formation in response to heat stress. These effects were seen in cells lacking either RNAi or CLRC H3K9 methyltransferase components (Ago1, Rik1 or Clr4); although we cannot rule out that each of these factors could have additional, independent functions impacting RNP granule assembly, the most parsimonious explanation for the common effects in all three deletion mutants is that they relate to the shared defect in heterochromatin. Moreover, the opposing effects of heterochromatin mutants on the frequency of granule formation in the presence versus absence of stress suggest that the granules arising in response to heterochromatin deficiency may be formed at the expense of canonical stress granules, suggesting competition for limiting factors. Stress granule formation is typically triggered by translational arrest, which leads to a sudden release of relatively protein-free and unfolded mRNAs from polysomes [66]. Interactions between these RNAs and aggregation-prone RNA-binding proteins leads to their phase separation into visible granules. We suggest that in the absence of heterochromatin, accumulating non-coding heterochromatic transcripts may also be bound by some of the same proteins. This would be consistent with our observation that in conditions where these proteins aggregate into stress granules, heterochromatic RNAs can be seen to co-localise. The increased occurrence of Pabp- and Cpn1-containing granules in heterochromatin-deficient cells in the absence of stress may also reflect the increased presence of heterochromatic RNPs in the cytoplasm, although it is also possible that they arise as a result of altered RNP homeostasis more broadly.

Our observation of interplay between heterochromatic silencing and stress granules is reminiscent of a similar phenomenon reported in human cells, where active siRNA-mediated silencing was found to antagonise stress granule formation. In that case, the effect was suggested to be explained by limiting factor Ago2 being required for both processes [67]. Here, it is absence of RNAi-directed heterochromatin formation that antagonises stress granule formation, which we suggest could reflect a role for factors such as Cpn1 in mopping up both transcripts released in response to stress, and those accumulating as a result of de-repression of heterochromatin. In simple terms, if RNA-binding proteins are drawn into interactions with accumulated heterochromatic transcripts independent of stress, this could deplete the pool of proteins available to bind translationally arrested RNAs in response to stress, explaining the reduction in heat-induced stress granule formation we observe in heterochromatin-deficient cells. Given that loss of heterochromatin is widely associated with ageing [68], such alterations in RNP homeostasis could also contribute to broader cellular dysregulation linked to age-related de-repression of heterochromatic domains.

The link between Cpn1 and regulation of heterochromatic transcripts is supported by our observation that absence of Cpn1 functionally impacts the accumulation of pericentromeric RNAs in heterochromatin-deficient cells, causing hyperaccumulation of these RNAs at centromeres and increased formation of DNA:RNA hybrids that likely explains the observed defect in pericentromeric heterochromatin establishment. This hyperaccumulation suggests that degradation of heterochromatic transcripts is impaired in *cpn1∆* cells. Interestingly, human CAPRIN1 was recently shown to promote selective transcript degradation, mediated by Xrn2, a normally nuclear ribonuclease that relocalises to the cytoplasm in a Caprin-dependent manner [59,69]. Here we present evidence consistent with possible conservation of this functional connection in fission yeast, since we show that: (1) inactivation of the Xrn2 ortholog, Dhp1, in *clr4∆* cells results in accumulation of pericentromeric transcripts in the cytoplasm; and (2) this cytoplasmic RNA accumulation is abolished in the absence of Cpn1. This suggests that Cpn1 is required upstream of Dhp1, possibly to facilitate delivery of RNAs for degradation. We also confirm that Dhp1, like Cpn1, is required for efficient heterochromatin establishment, consistent with previous studies [22,23]. In comparison to Cpn1, loss of Dhp1 appears to have a greater impact on heterochromatin maintenance, including at facultative heterochromatin islands and HOODs, consistent with Dhp1 having additional, Cpn1-independent functions in promoting heterochromatin formation. Conversely, nucleases other than Dhp1 may also contribute Cpn1-dependent degradation[70].

An outstanding question from our work is whether Cpn1 itself interacts directly with heterochromatic transcripts. Cpn1 is predicted to be an RNA-binding protein, and we found that the potentially RNA-binding RG/RGG-rich region is important for Cpn1 function in promoting efficient heterochromatin establishment. However, we did not detect stable association of Cpn1 with *cen*(*dg*) transcripts by RNA-IP, suggesting that any interaction may be weak/transient. Nevertheless, their functional association is strongly supported by our imaging analyses that revealed co-localisation of Cpn1 with *cen*(*dg*) transcripts in heterochromatin-deficient cells, both in the nucleus in the absence of stress, and in the cytoplasm, either in response to stress, or when Dhp1-mediated degradation is impaired. Although predominantly a cytoplasmic protein, the capacity for Cpn1 to localise to the nucleus is consistent with previous findings that both Cpn1, and its binding partner Nxt3, accumulate in the nucleus following treatment of cells with leptomycin B, which inhibits CRM1-dependent nuclear export [58]. This suggests that, like many RNA-binding proteins, they have the capacity to shuttle between the cytoplasm and the nucleus, and so could play a role in nucleocytoplasmic transport. Consistent with this, the human ortholog of Nxt3, G3BP1, has been shown to undergo phosphorylation-dependent nuclear import [70]. Moreover, our findings also reveal the capacity for heterochromatic transcripts to be translocated from the nucleus to the cytoplasm. Although not previously reported in *S. pombe*, cytosolic localisation of heterochromatic transcripts is not without precedent, since in mouse pancreatic cancer models, aberrantly expressed centromeric satellite repeat RNAs have been seen to accumulate in the cytoplasm [71]. It may be that detection of *cen(dg)* RNAs in the cytoplasm in *S. pombe* is only possible in conditions where they are aggregated (via their associated RBPs) in stress granules, or their normal rapid degradation otherwise suppressed. Finally, we have also shown that this cytoplasmic accumulation of *cen*(*dg*) transcripts, both in response to stress and upon Dhp1 inactivation, is dependent on Cpn1, pointing to a role for Cpn1 in facilitating heterochromatic transcript export from the nucleus. Therefore, taken together, our findings suggest a model whereby Cpn1 functionally associates with heterochromatic transcripts to help target them for Dhp1-mediated cytoplasmic degradation (S9 Fig).

Human CAPRIN1 is increasingly being recognised as a protein of interest in several clinical contexts. For example, it contributes to antiviral responses, and several viruses, including Zika virus, have been found to hijack CAPRIN1 to inhibit stress granule formation and thus promote viral replication [72]. Moreover, CAPRIN1 also plays important roles in brain development, regulating the transport and translation of mRNAs required for synaptic plasticity. Deficiency of CAPRIN1 has been linked to autism spectrum disorders [73] and long-term memory impairment [74], while a specific missense mutation (P512L) has recently been implicated in early-onset ataxia and neurodegeneration [75]. In addition, changes in CAPRIN1 expression and localisation have also been linked to cancer; in particular, the recent detection of CAPRIN1 on the cell membrane surface in many solid cancers, but not normal tissues, makes it an attractive novel target for cancer therapeutics [76]. Despite their potential importance, the roles of CAPRIN1 outside of stress granules remain poorly understood; the identification of a Caprin family protein in fission yeast opens new opportunities to dissect the molecular mechanisms underpinning the cellular functions of these proteins, as well as unveiling a novel connection between RNP homeostasis and heterochromatin assembly.

## Materials and methods

### Yeast strains and methods

Strains used in this study are listed in S3 Table. Standard procedures were used for growth and genetic manipulations. Cultures were grown at 32^o^C unless otherwise stated, in either rich medium (YES) or minimal media (PMG). Genomic integrations for gene deletion and epitope-tagging were achieved by homologous recombination using PCR-based modules consisting of a resistance cassette flanked by 80 bp sequences homologous to the target locus. To generate the *cpn1*^*R6A*^ mutant, the six arginines in the RGG motif were mutated to alanine by CRISPR/Cas9 editing as previously described [77]. Genomic modifications were verified by sequencing. Overexpression plasmids pSP102-Sir2, pSP102-Clr3 and pSP102-Sir2-Clr3 were described previously [78]. The minichromosome establishment assay was carried out as described previously [32]. For cross-based establishment assays, random spore analysis was employed. Strains bearing different gene deletions marked with *ura4*^*+*^ were crossed, and the products plated onto selective media containing 1g/L 5-FOA and low (1/10^th^) adenine to directly select for desired recombinant genotypes (and against parental genotypes) prior to counting of red/white colonies.

### Live cell imaging

Visualisation of fluorescently-tagged proteins in living cells was performed on log phase cultures grown at 32°C in PMG and, where indicated, pre-treated as follows: heat shock, 20 min incubation at 42°C; glucose starvation, 20 min incubation in medium lacking glucose. Images were acquired using a Nikon Ti2 inverted microscope equipped with a 100x 1.49 NA Apo TIRF objective and a Teledyne Photometrics Prime 95B camera. Images were acquired with NIS-elements (version 5.1), with 25 z-stacks taken at 0.2µm intervals, deconvolved with AutoQuant X3 software and subsequently exported to ImageJ for analysis. Quantification of foci number/area was achieved by intensity threshold segmentation followed by application of the ‘analyse particles’ function of ImageJ.

### smRNA FISH

smRNA FISH was performed essentially as described previously [79]. Briefly, 2×10^8^ cells were fixed with 4% paraformaldehyde for 30 min, or 2% paraformaldehyde for 30 min for simultaneous imaging of GFP-tagged Pabp or Cpn1. Cells were washed with Buffer B (1.2 M sorbitol, 0.1M potassium phosphate buffer pH 7.5), resuspended in spheroplast buffer (1.2 M sorbitol, 0.1 M potassium phosphate, 20 mM vanadyl ribonuclease complex [NEB, S1402S], 20 μM beta-mercaptoethanol) and digested with 0.002% 100 T zymolyase (US Biological, Z1005) for approximately 45–75 min. Cells were washed with Buffer B, resuspended in 1 ml of 0.01% Triton X-100 in 1x PBS for 20 min, washed again with Buffer B, and resuspended in 10% formamide/2x SSC buffer. For hybridization of probes, approximately 20–25ng of CAL Fluor red 610 probes targeting *cen(dg)* repeat sequences (Stellaris, Biosearch Technologies) were mixed with 2 μl each of yeast tRNA (Life Technologies) and Salmon sperm DNA (Life Technologies) per reaction. Sequences of the *cen(dg)* probes are given in S5 Table. The probe solution was mixed with Buffer F (20% formamide, 10 mM sodium-phosphate buffer pH 7.2; 45 μl per reaction), heated at 95°C for 3 min, and allowed to cool to room temperature before mixing with Buffer H (4x SSC, 4 mg/ml acetylated BSA, 20 mM vanadyl ribonuclease complex; 50 μl per reaction). Digested cell samples were divided into two reactions, each of which was resuspended in 100 μl of this hybridization solution and incubated at 37°C overnight. Cells were subsequently washed sequentially with: 10% formamide/2x SSC; 0.1% Triton X-100/2x SSC; 2x SSC; 1x SSC; and 1x PBS. Cell pellets were mixed with VECTASHIELD Vibrance Antifade Mounting Medium with DAPI (Vector Laboratories) and mounted on poly-L-Lysine slides with 1.5mm glass coverslips. Imaging was performed using a Nikon Ti2 inverted microscope equipped with a 100x 1.49 NA Apo TIRF objective and a Teledyne Photometrics Prime 95B camera. Images were acquired with NIS-elements (version 5.1), with 25 z-stacks taken at 0.2µm intervals, further deconvolved with AutoQuant X3 software and subsequently exported to ImageJ for analysis. Quantification of RNA foci intensity was achieved by intensity threshold segmentation followed by application of the ‘analyse particles’ function of ImageJ.

### RT-qPCR

Total RNA was extracted from 1 × 10^7^ cells in exponential growth phase using the Masterpure Yeast RNA Purification Kit (Epicentre) according to the manufacturer’s instructions. 1 μg of total RNA was treated with TURBO DNase (Ambion) for 1 h at 37°C, then reverse transcribed using random hexamers (Roche) and Superscript III reverse transcriptase (Invitrogen) according to the manufacturer’s instructions. cDNA was quantified by qPCR using LightCycler 480 SYBR Green (Roche) and primers listed in S4 Table. In all cases, histograms represent three biological replicates and error bars represent one S.D.

### RNA-seq

Total RNA was isolated as above and provided to Azenta Life Sciences for polyA selection, library preparation, and sequencing using an Illumina Novaseq X, generating a minimum of 30 million 150 bp paired-end reads per sample. Three biological replicates for wild-type and *cpn1∆* were processed in parallel. Adapters were removed from raw reads using Cutadapt (v4.8) [80] and the resulting trimmed reads were aligned to the *S. pombe* reference genome (ASM294v2.27) and a previously generated GTF file using STAR (v2.7.10b) [81]. FeatureCounts from the Rsubread (v2.16.0) R package [82] was used to generate the raw count matrix which was then used as input for differential expression analysis with the edgeR (v3.42.4) R package [83]. Genes with a log2 fold change (Log2FC) greater than 1 or less than − 1, and a False Discovery Rate (FDR) less than 0.05, were considered differentially expressed.

### ChIP and DRIP

ChIP and DRIP experiments were performed essentially as described previously [84]. Briefly, 2.5 × 10^8^ cells per IP were fixed in 1% formaldehyde for 15 min at room temperature. Cells were lysed using a bead beater (Biospec products, 2 x 2 min) and sonicated using a Bioruptor (Diagenode) for a total of 20 min (30 s on/30 s off on ‘high’ power). Immunoprecipitation was then performed overnight at 4°C, using 1 μl per IP of monoclonal anti-H3K9me2 (5.1.1 [85]) for ChIP, or 1 μl of monoclonal S9.6 anti-DNA-RNA hybrid (MABE1095, MerckMilipore) for DRIP. Immunoprecipitated DNA was recovered using Chelex-100 resin (BioRad), and quantified by qPCR using LightCycler 480 SYBR Green (Roche) and primers listed in S4 Table. Relative enrichments were calculated as the ratio of product of interest to control product (*act1*^*+*^) in IP over input. In all cases, histograms represent three biological replicates and error bars represent one S.D.

### Immunoprecipitation

For IP-western, cultures were grown to mid-log phase in YES and 3 x 10^8^ cells were harvested, washed and resuspended in lysis buffer (50 mM Hepes pH 7.5, 150 mM NaCl, 5 mM EDTA, 0.1% NP-40, 1 mM PMSF, 1× yeast protease inhibitor cocktail [Sigma]). Cells were lysed using a bead beater (Biospec products, 2 x 2 min), DTT was added to a final concentration of 500 μM, and the supernatant was recovered by 2 x 15 min centrifugation at 17,000 rcf at 4°C. Extracts were pre-cleared by incubation with pre-equilibrated protein G agarose for 1 hour at 4°C, and then incubated with pre-equilibrated protein G agarose plus anti-FLAG M2 (F3165, Merck, 1 μl/IP), or anti-GFP (A-11122, ThermoFisher, 5 ul/IP), for 3 hours at 4°C. The beads were washed three times with lysis buffer, and proteins eluted in 2x SDS sample buffer (50mM Tris-HCl pH 6.8, 2mM EDTA, 10% glycerol, 2% SDS, 2% β-Mercaptoethanol, 0.03% Bromophenol Blue) by boiling for 5 min. For analysis, proteins were separated by SDS-PAGE and transferred onto 0.45μm pore Protran nitrocellulose membrane (GE Healthcare) using semi-dry transfer apparatus (Hoeffer). Membranes were probed with mouse anti-FLAG M2 (F3165, Merck, 1:1000 dilution) or rabbit anti-GFP (A-11122, ThermoFisher, 1:1000 dilution). Secondary antibodies were IRDye conjugated anti-mouse or anti-rabbit (926-32210 or 926-68171, Li-Cor, 1:20,000 dilution) and imaging was performed using the Odyssey CLx Imaging System (LI-COR Biosciences).

Immunoaffinity purifications for mass spectrometry analysis were performed essentially as described previously [86]. Briefly, 500 ml cultures grown to a cell density of 10^8^ cells/ml in 4× concentrated YES media were milled in solid phase, and the cell powder solubilised in 10ml lysis buffer (50 mM HEPES–NaOH pH 7.5, 150 mM KCl, 0.1% NP-40, 0.2 mM PMSF, 0.2mM benzamidine, 1× EDTA-free protease inhibitor cocktail [Roche]). Following clarification by two rounds of centrifugation, immunoprecipitations performed by incubating with 8 μl Protein G Dynabeads (Novex) and 12 μl anti-FLAG antibody (F3165, Merck) per sample for 90 min at 4°C. The immunoprecipitated material was washed with lysis buffer, incubated in lysis buffer containing 2 mM MgCl_2_ and 500 U of Benzonase nuclease (Novagen) for 15 min at 4°C, washed again with lysis buffer, and then eluted by incubation with 50 μl of 0.1% Rapigest (Waters) in 50 mM Tris-HCl pH 8.0 for 10 min at 50°C prior to LC-MS/MS analysis.

RNA-IPs were performed as above but with modified lysis buffer (50 mM HEPES–NaOH pH 7.5, 150 mM NaCl, 1mM MgCl_2_, 0.1% NP-40, 5 mM DTT, 0.5 mM PMSF, 1× EDTA-free protease inhibitor cocktail [Roche], 0.2U/μl RNAsin Ribonuclease Inhibitor [Promega]), and excluding Benzonase treatment. After initial washes, immunoprecipitated material was resuspended in RNA extraction buffer (25 mM Tris–HCl pH 7.5, 5 mM EDTA pH 8, 50 mM NaCl, 0.5% SDS) with 200 ng/ml proteinase K and incubated at 37°C for 2 h. RNA was extracted with phenol:chloroform, precipitated with ethanol and 1 μl glycogen, and analysed by RT-qPCR.

### Mass spectrometry

IP eluate was reduced with 25 mM DTT at 80°C for 1 min, then denatured by addition of urea to 8M. Sample was applied to a Vivacon 30k MWCO spin filter (Sartorius, VN01H21) and centrifuged at 12,500 g for 15 min. Protein retained on the column was then alkylated with 100 μl of 50 μM iodoacetamide (IAA) in buffer A (8 M urea,100 mM Tris pH 8.0) in the dark at RT for 20 min. The column was then centrifuged as before, and washed with 100 μl buffer A, then with 2 x 100 μl volumes of 50 mM ammonium bicarbonate (ABC). 3 μg/μl trypsin (Pierce, 90057) in 0.5 mM ABC was applied to the column and incubated at 37° for 16 hr. Digested peptides were then spun through the filter, acidified with trifluoroacetic acid (TFA) to pH <= 3, loaded onto manually-prepared and equilibrated C_18_ reverse-phase resin stage tips (Sigma, 66883-U) [87], washed with 100 μl 0.1% TFA, and stored at -20°C.

For MS analysis, peptides were eluted in 40 μl of 80% acetonitrile in 0.1% TFA and concentrated down to 2 μl by vacuum centrifugation (Concentrator 5301, Eppendorf). The peptide sample was then prepared for LC-MS/MS analysis by diluting it to 5 μl by 0.1% TFA. LC-MS-analyses were performed on an Orbitrap Exploris 480 Mass Spectrometer (Thermo Scientific) coupled on-line, to an Ultimate 3000 RSLCnano Systems (Dionex, Thermo Fisher Scientific). Peptides were separated on a 50 cm EASY-Spray column (Thermo Scientific), which was assembled on an EASY-Spray source (Thermo Scientific) and operated at 50°C. Mobile phase A consisted of 0.1% formic acid in LC-MS grade water and mobile phase B consisted of 80% acetonitrile and 0.1% formic acid. Peptides were loaded onto the column at a flow rate of 0.3 μl per min and eluted at a flow rate of 0.25 μl per min according to the following gradient: 2 to 40% mobile phase B in 150 min, and then to 95% in 11 min. Mobile phase B was retained at 95% for 5 min and returned back to 2% a minute after until the end of the run (190 min). FTMS spectra were recorded at 120,000 resolution (scan range 350-1500 m/z) with an ion target of 3.0×10^6^ and maximum injection time of 50 ms. For the MS2 the resolution was set at 15,000 with ion target of 8.0×10^4^ and HCD fragmentation [88] with normalized collision energy of 30. The isolation window in the quadrupole was 1.4 Thomson. Only ions with charge between 2 and 6 were selected for MS2.

The MaxQuant software platform [89] version 1.6.1.0 was used to process the raw files and search was conducted against the *Schizosaccharomyces pombe* complete/reference proteome (Pombase, released in July 2017), using the Andromeda search engine [90]. For the first search, peptide tolerance was set to 20 ppm while for the main search peptide tolerance was set to 4.5 pm. Isotope mass tolerance was 2 ppm and maximum charge to 7. Digestion mode was set to specific with trypsin allowing a maximum of two missed cleavages. Carbamidomethylation of cysteine was set as fixed modification. Oxidation of methionine was set as variable modification. Absolute protein quantification was performed as described previously [91]. Peptide and protein identifications were filtered to 1% FDR.

## Supporting information

S1 Fig Efficiency of heterochromatin re-establishment varies with genetic background, but is consistent for the same background. (A) Proportions of red (*cen1:ade6^+^* silenced) versus white (*cen1:ade6^+^* expressed) colonies in the wild-type progeny of crosses between the indicated parental strains, based on analysis of n colonies. (B) Proportions of red (*cen1:ade6*^*+*^ silenced) versus white (*cen1:ade6*^*+*^ expressed) colonies in the wild-type progeny of crosses between three independent *rik1∆* and *ago1∆* strains, based on analysis of *n* colonies.
(TIF)

S2 Fig Cpn1 is not required for silencing at heterochromatin islands or most HOODs. (A) RT-qPCR analysis of indicated transcript levels relative to *act1^+^*, normalized to wild-type. (B) ChIP-qPCR analysis of H3K9me2 levels at indicated HOODs, relative to *act1*^*+*^, normalised to wild-type. In all cases data are averages of three biological replicates; dots represent individual data points and error bars represent one SD. Relative to wild-type, asterisks denote p ≤ 0.05 (*), or p ≤ 0.01 (**), from Student’s t-test analysis.
(TIF)

S3 Fig Mkt1 but not Cpn1 interacts with centromeric transcripts. RNA-immunoprecipitation (RNA-IP) analysis of transcripts associated with FLAG-tagged Cpn1 or Mkt1 (wild-type or cpn1∆ backgrounds), under native conditions. IP enrichments are shown relative to input. Data are averages of three biological replicates; dots represent individual data points and error bars represent one SD. Relative to untagged control, asterisks denote p ≤ 0.05 (*), or p ≤ 0.001 (***), from Student’s t-test analysis.
(TIF)

S4 Fig Absence of Cpn1 is associated with increased heterochromatin formation at subtelomeres. (A) ChIP-qPCR analysis of H3K9me2 levels at the indicated loci relative to *act1^+^*, normalised to wild-type. Data are averages of three biological replicates; dots represent individual data points and error bars represent one SD. Asterisks denote p ≤ 0.05 (*), or p ≤ 0.01 (**), from Student’s t-test analysis. (B) Genome browser views of ChIP-seq data taken from Verrier et al. [48] showing H3K9me2 levels in wild-type cells at Tel1L, Tel1R, and Tel2R, on log2 scale. Genome annotation is shown below with loci analysed in A highlighted to show their position relative to normal H3K9me2 boundaries; underlining indicates loci found to be >1.5-fold down-regulated, and bold, loci found to be >2-fold down-regulated, from RNA-seq analysis.
(TIF)

S5 Fig GFP-tagged Cpn1, Nxt3 and Ubp3 are stable and functional. (A) Western blot analysis of affinity-purified GFP-tagged Nxt3, Ubp3, Cpn1, and Cpn1R6A. An untagged (no tag) wild-type strain is included as a control for antibody specificity. (B) Proportions of red (*cen1:ade6*^*+*^ silenced) versus white (*cen1:ade6*^*+*^ expressed) colonies in the otherwise wild-type progeny of *rik1∆* x *ago1∆* crosses performed in the indicated genetic backgrounds, based on analysis of *n* colonies.
(TIF)

S6 Fig Cpn1 expression levels are unaffected by deletion of heterochromatin factors. Western blot analysis of Cpn1-GFP levels in the indicated strains. An untagged (no tag) wild-type strain is included as a control for antibody specificity; * indicates non-specific signal.
(TIF)

S7 Fig Disruption of heterochromatin is associated with reduced formation of Pabp-containing RNP granules in heat shock but not glucose starvation. (A) Quantification of the mean Pabp-mRFP focus area, and mean total Pabp-mRFP foci area per cell, in cells exposed to 42^°^C heat shock for 20 min, based on analysis of *n* foci and cells, respectively, from the imaging analysis shown in Fig 5D. Asterisks denote p ≤ 0.001 (***) from Student’s t-test analysis. (B) Quantification of the same parameters as in (A), as well as the mean number of Pabp foci per cell, in cells exposed to 20 min glucose starvation, from the imaging analysis shown in (C). (C) Representative images from live-cell imaging of Pabp-mRFP (red) in the indicated genetic backgrounds in cells exposed to 20 min glucose starvation. Bar indicates 6 µm.
(TIF)

S8 Fig Absence of Cpn1 results in increased accumulation of pericentromeric transcripts at centromeres in ago1∆ cells. (A) Representative images from simultaneous analysis of *cen(dg)* RNA by smRNA-FISH, and Pabp-GFP, in *ago1∆* cells either unstressed (+ glucose) or stressed by 20 min of glucose starvation (- glucose). Bar indicates 6 µm, and arrow heads highlight examples of co-localisation (Pearson’s correlation coefficient, *R*, is indicated, and inserts show 5x magnification). (B) Representative images from smRNA-FISH analysis of *cen(dg)* RNA in *ago1∆* and *ago1∆ cpn1∆* cells. In both A and B, data are representative of at least three independent experiments. (C) Quantification of the mean signal intensity for nuclear *cen(dg)* RNA foci in *ago1∆ cpn1∆* relative to *ago1∆* cells, from the smRNA-FISH analysis shown in B. (D) RT-qPCR analysis of total cellular levels of *cen*(*dg*) RNA relative to *act1*^*+*^, in *ago1∆ cpn1∆* relative to *ago1∆* cells. RT-qPCR data are averages of three biological replicates; dots represent individual data points. In all cases, error bars represent one SD, and asterisks denote p ≤ 0.05 (*), or p ≤ 0.001 (***), from Student’s t-test analysis.
(TIF)

S9 Fig Model for impact of Cpn1 on pericentromeric transcript regulation. Cpn1 associates with pericentromeric RNAs to target them for Dhp1-dependent degradation. In comparison to wild-type cells, in *clr4∆* cells there is increased pericentromeric transcript accumulation, leading to greater Cpn1 association; this reduces the free Cpn1 pool available for stress granule formation in response to stress. Absence of Cpn1 leads to reduced targeting for Dhp1-mediated degradation, and hence hyperaccumulation of pericentromeric transcripts in the nucleus. Absence of Dhp1 results in some accumulation of pericentromeric transcripts in the cytoplasm, suggesting that Cpn1 may help to promote their export for cytoplasmic degradation.
(TIF)

S1 Table Transcripts upregulated in *cpn1*∆ cells (log2FC > 1; FDR < 0.05). (DOCX)

S2 Table Transcripts downregulated in *cpn1*∆ cells (log2FC < -1; FDR < 0.05). (DOCX)

S3 Table Yeast strains. (DOCX)

S4 Table qPCR primers. (DOCX)

S5 Table smRNA FISH probes. (DOCX)
