# Supplementary figures and images for: Fission yeast Caprin protein is required for efficient heterochromatin establishment
Source: PLoS Genet. 2025 Mar 10;21(3):e1011620. doi: 10.1371/journal.pgen.1011620 (PMC11918387; doi:10.1371/journal.pgen.1011620)

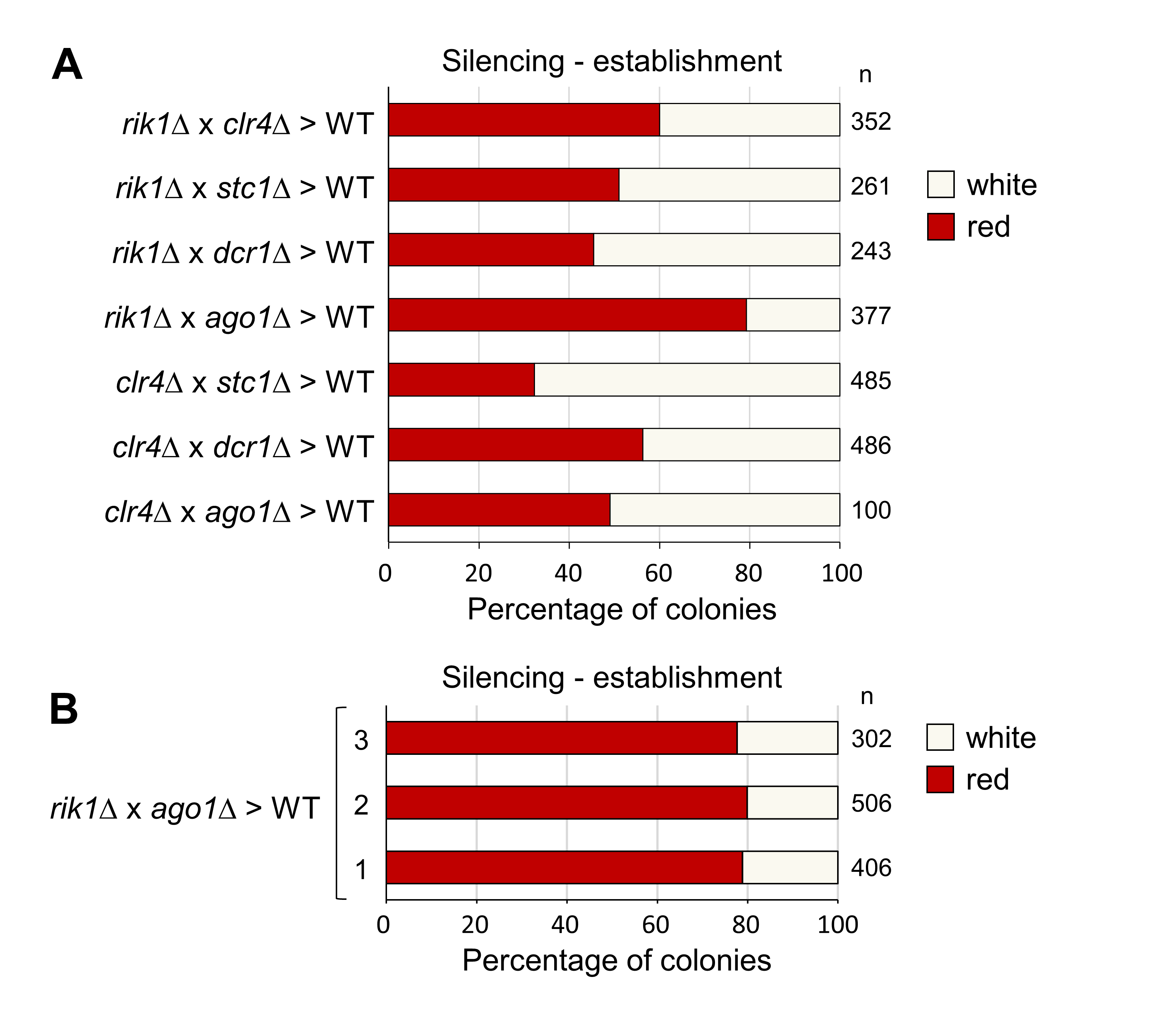

Supplement: S1 Fig — (A) Proportions of red (cen1:ade6+ silenced) versus white (cen1:ade6+ expressed) colonies in the wild-type progeny of crosses between the indicated parental strains, based on analysis of n colonies. (B) Proportions of red (cen1:ade6+ silenced) versus white (cen1:ade6+ expressed) colonies in the wild-type progeny of crosses between three independent rik1∆ and ago1∆ strains, based on analysis of n colonies. (TIF) [file pgen.1011620.s001.tif]

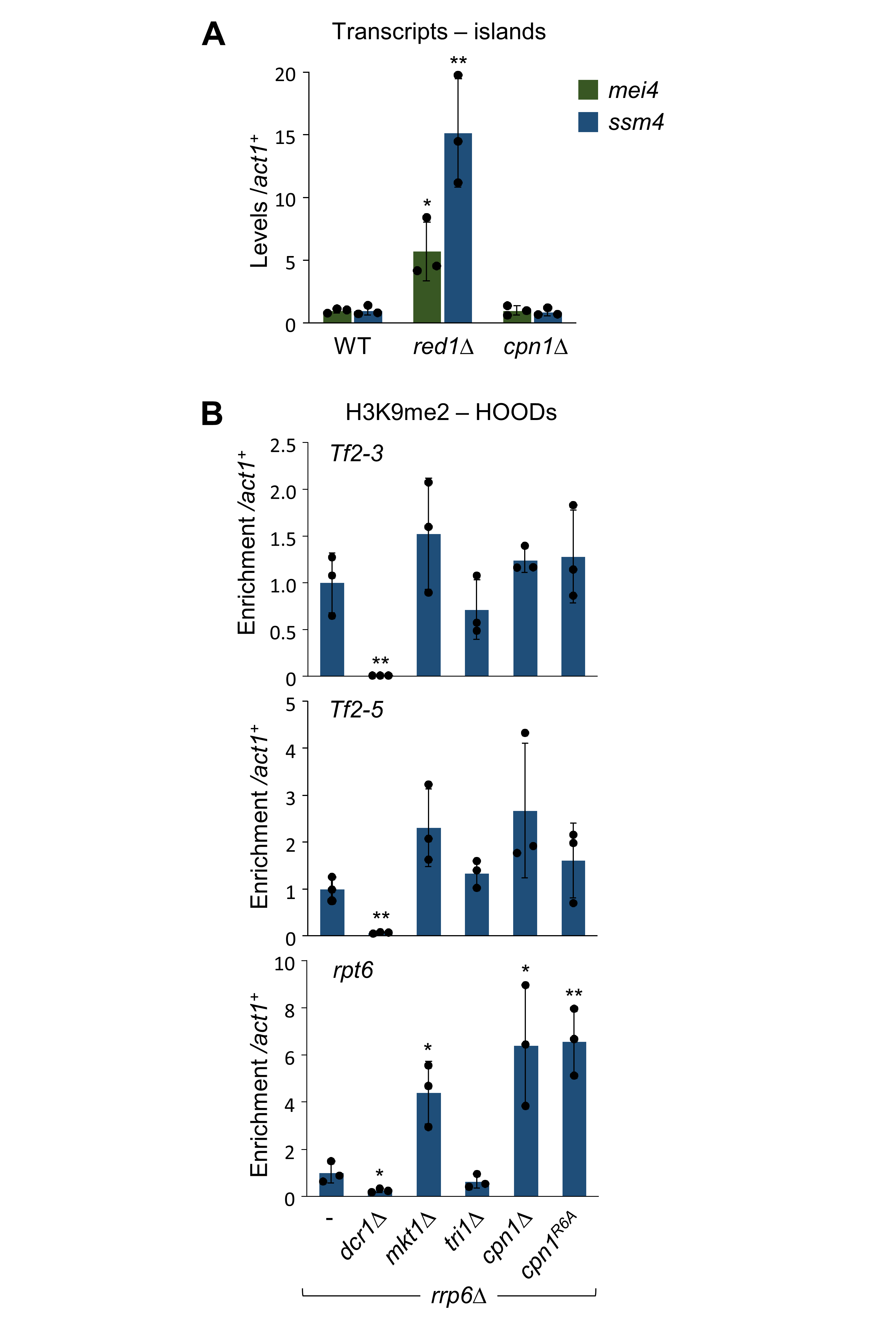

Supplement: S2 Fig — (A) RT-qPCR analysis of indicated transcript levels relative to act1+, normalized to wild-type. (B) ChIP-qPCR analysis of H3K9me2 levels at indicated HOODs, relative to act1+, normalised to wild-type. In all cases data are averages of three biological replicates; dots represent individual data points and error bars represent one SD. Relative to wild-type, asterisks denote p ≤ 0.05 (*), or p ≤ 0.01 (**), from Student’s t-test analysis. (TIF) [file pgen.1011620.s002.tif]

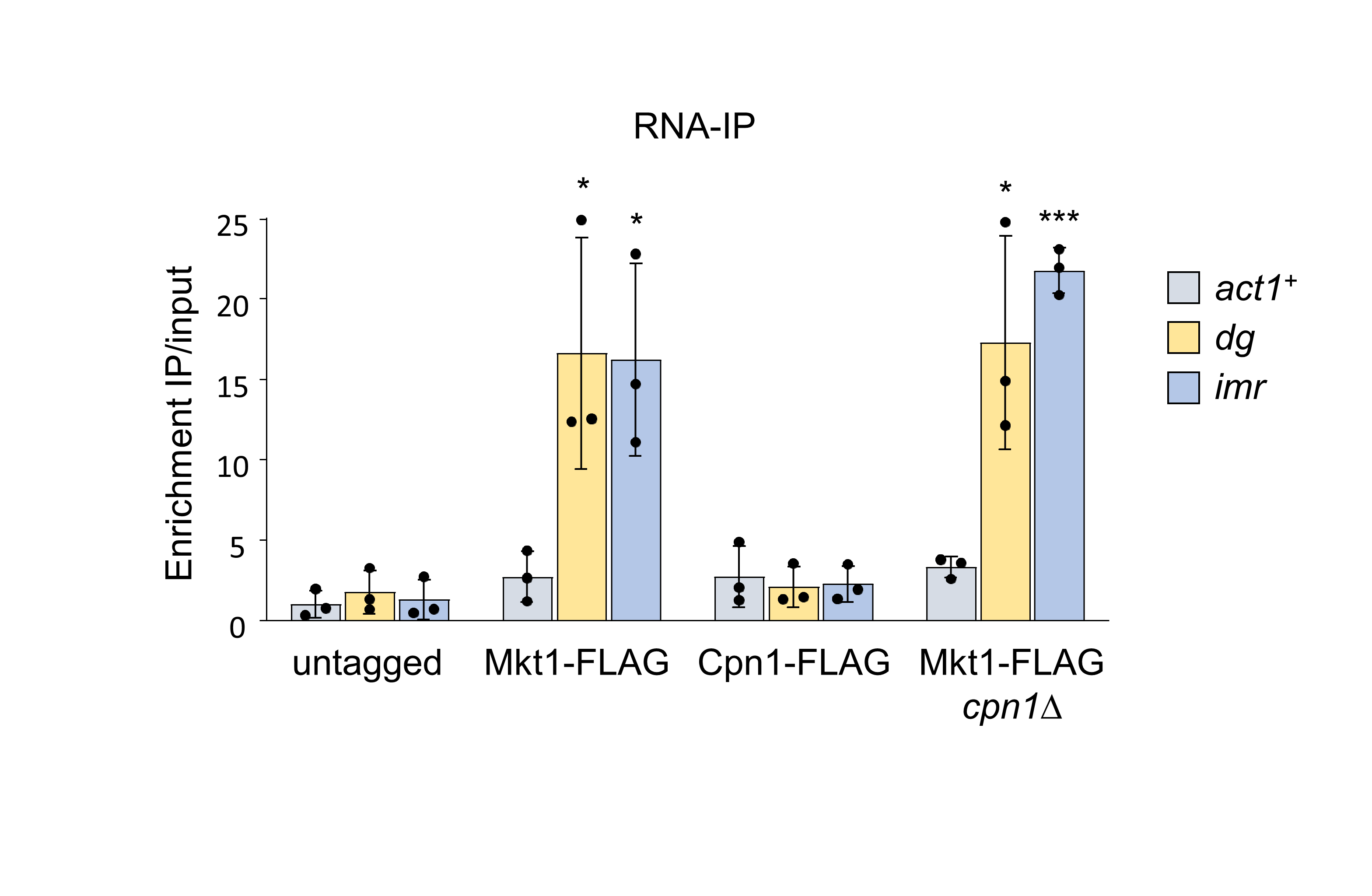

Supplement: S3 Fig — RNA-immunoprecipitation (RNA-IP) analysis of transcripts associated with FLAG-tagged Cpn1 or Mkt1 (wild-type or cpn1∆ backgrounds), under native conditions. IP enrichments are shown relative to input. Data are averages of three biological replicates; dots represent individual data points and error bars represent one SD. Relative to untagged control, asterisks denote p ≤ 0.05 (*), or p ≤ 0.001 (***), from Student’s t-test analysis. (TIF) [file pgen.1011620.s003.tif]

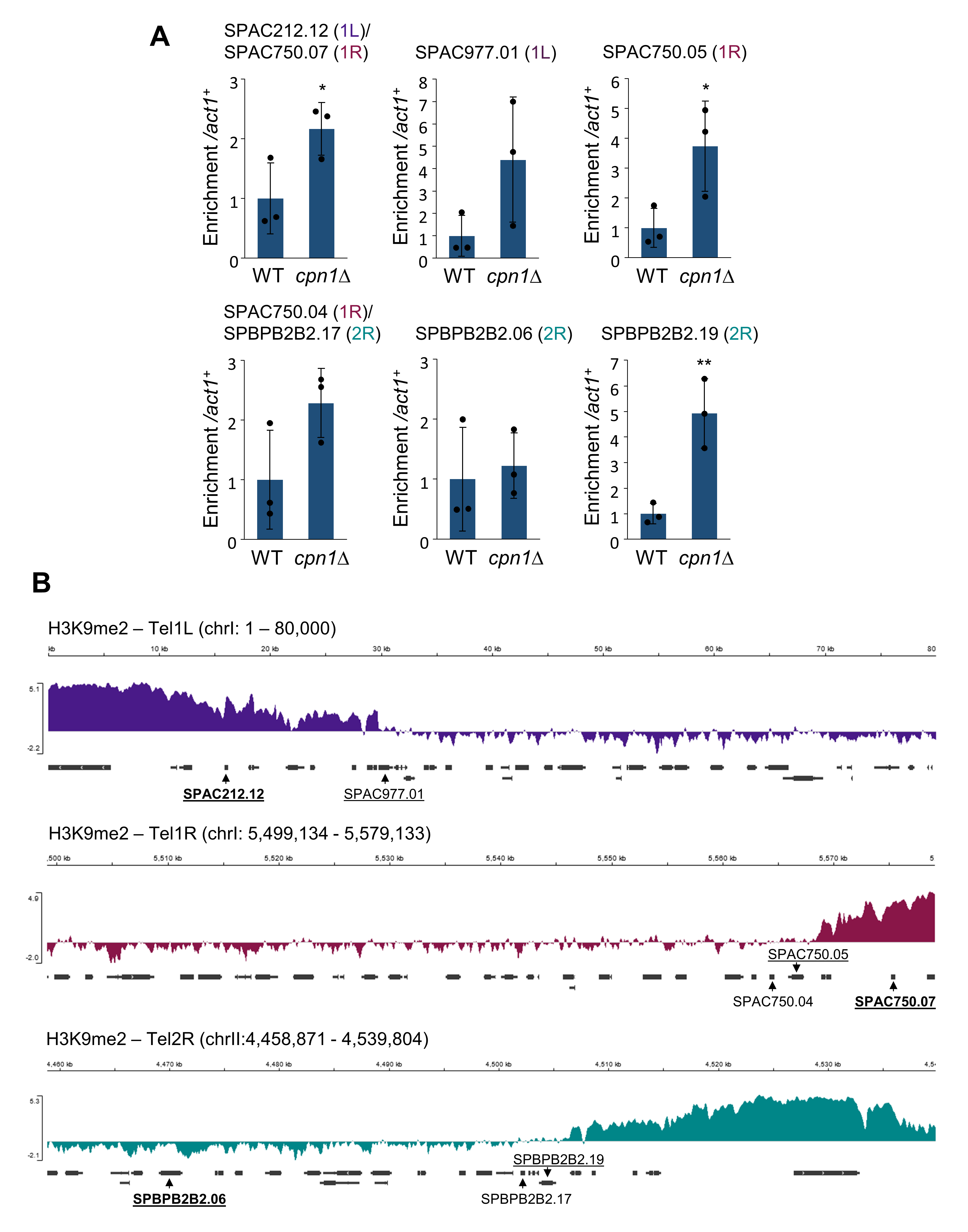

Supplement: S4 Fig — (A) ChIP-qPCR analysis of H3K9me2 levels at the indicated loci relative to act1+, normalised to wild-type. Data are averages of three biological replicates; dots represent individual data points and error bars represent one SD. Asterisks denote p ≤ 0.05 (*), or p ≤ 0.01 (**), from Student’s t-test analysis. (B) Genome browser views of ChIP-seq data taken from Verrier et al. [48] showing H3K9me2 levels in wild-type cells at Tel1L, Tel1R, and Tel2R, on log2 scale. Genome annotation is shown below with loci analysed in A highlighted to show their position relative to normal H3K9me2 boundaries; underlining indicates loci found to be >1.5-fold down-regulated, and bold, loci found to be >2-fold down-regulated, from RNA-seq analysis. (TIF) [file pgen.1011620.s004.tif]

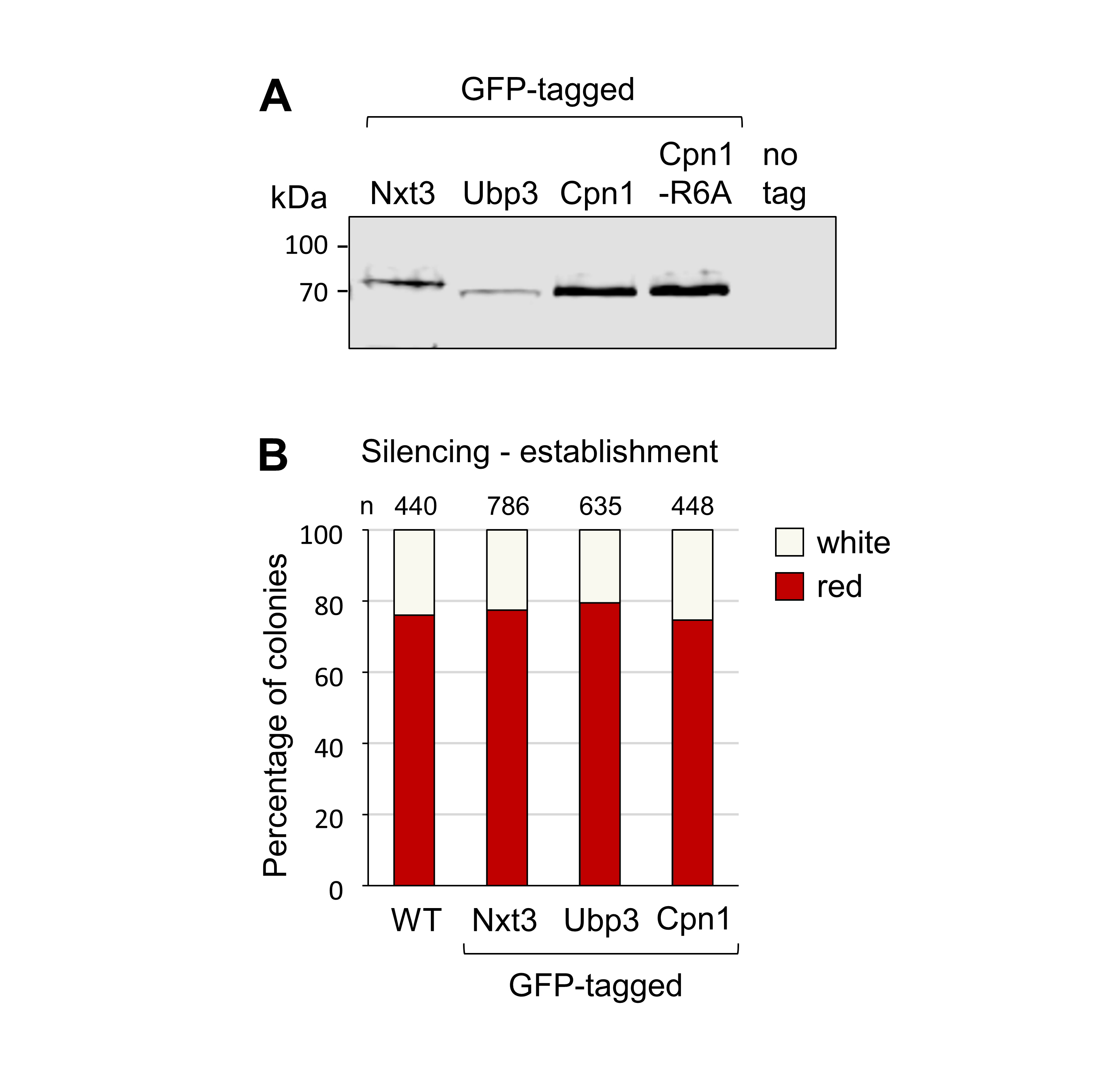

Supplement: S5 Fig — (A) Western blot analysis of affinity-purified GFP-tagged Nxt3, Ubp3, Cpn1, and Cpn1R6A. An untagged (no tag) wild-type strain is included as a control for antibody specificity. (B) Proportions of red (cen1:ade6+ silenced) versus white (cen1:ade6+ expressed) colonies in the otherwise wild-type progeny of rik1∆ x ago1∆ crosses performed in the indicated genetic backgrounds, based on analysis of n colonies. (TIF) [file pgen.1011620.s005.tif]

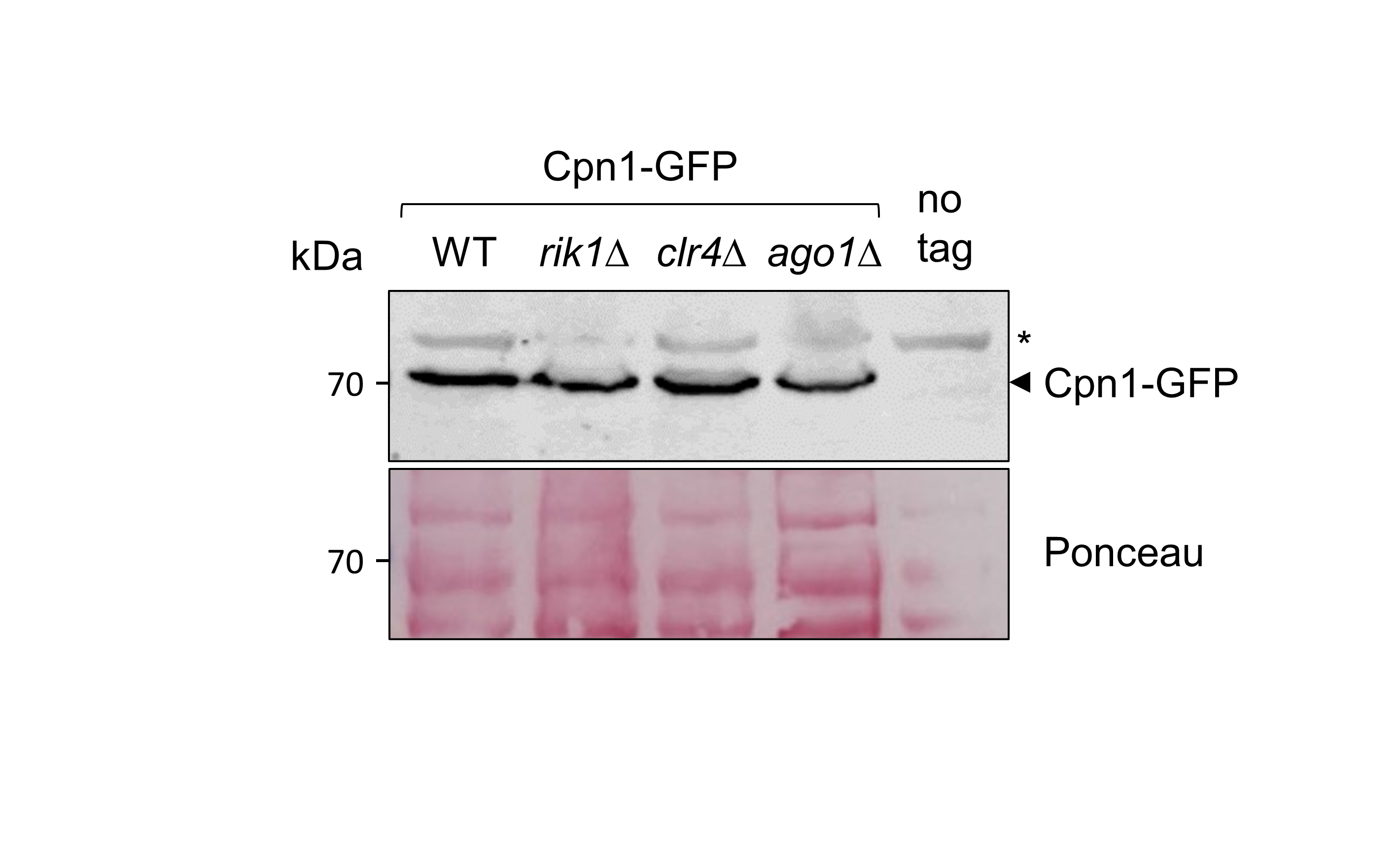

Supplement: S6 Fig — Western blot analysis of Cpn1-GFP levels in the indicated strains. An untagged (no tag) wild-type strain is included as a control for antibody specificity; * indicates non-specific signal. (TIF) [file pgen.1011620.s006.tif]

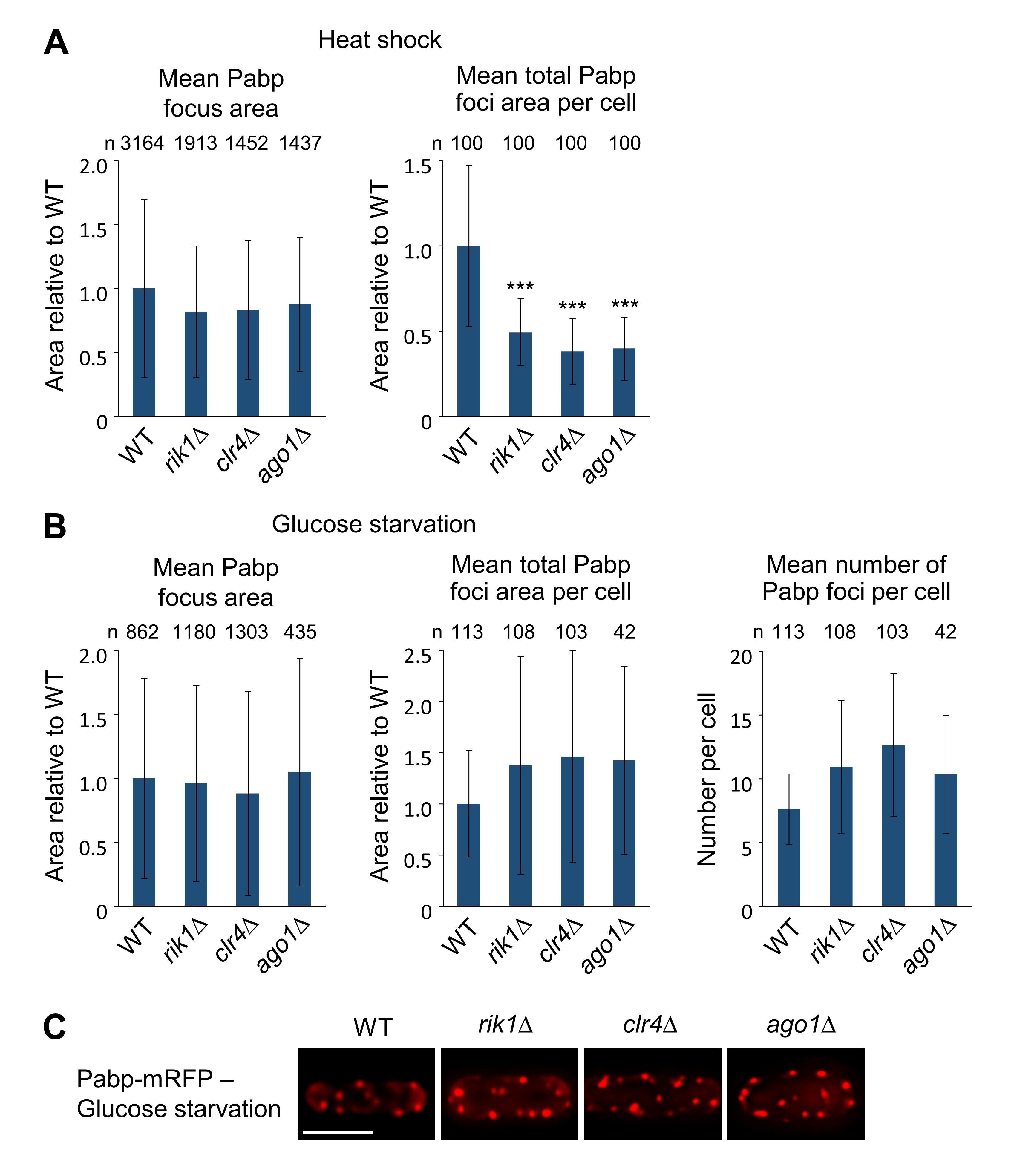

Supplement: S7 Fig — (A) Quantification of the mean Pabp-mRFP focus area, and mean total Pabp-mRFP foci area per cell, in cells exposed to 42°C heat shock for 20 min, based on analysis of n foci and cells, respectively, from the imaging analysis shown in Fig 5D. Asterisks denote p ≤ 0.001 (***) from Student’s t-test analysis. (B) Quantification of the same parameters as in (A), as well as the mean number of Pabp foci per cell, in cells exposed to 20 min glucose starvation, from the imaging analysis shown in (C). (C) Representative images from live-cell imaging of Pabp-mRFP (red) in the indicated genetic backgrounds in cells exposed to 20 min glucose starvation. Bar indicates 6 µm. (TIF) [file pgen.1011620.s007.tif]

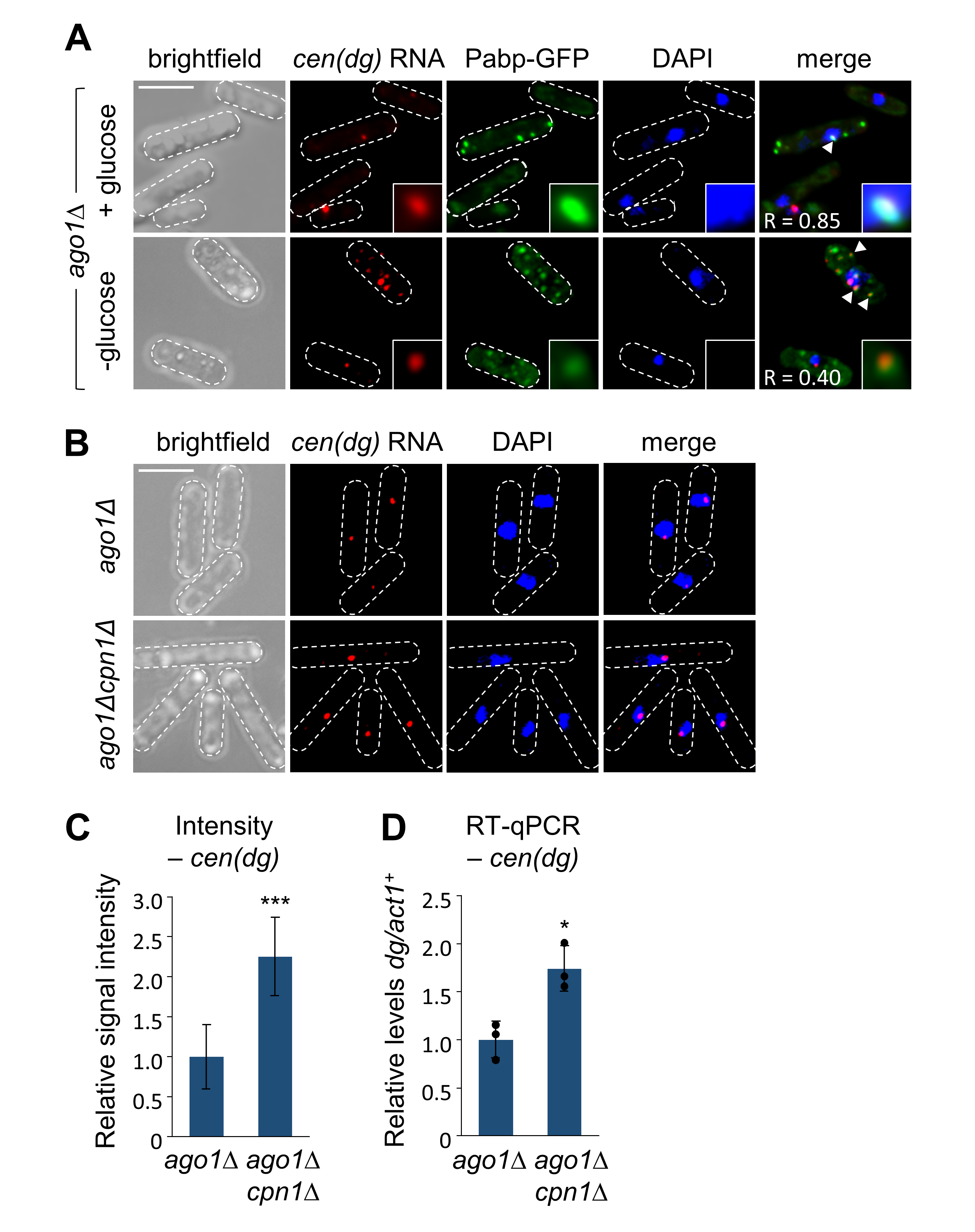

Supplement: S8 Fig — (A) Representative images from simultaneous analysis of cen(dg) RNA by smRNA-FISH, and Pabp-GFP, in ago1∆ cells either unstressed (+ glucose) or stressed by 20 min of glucose starvation (- glucose). Bar indicates 6 µm, and arrow heads highlight examples of co-localisation (Pearson’s correlation coefficient, R, is indicated, and inserts show 5x magnification). (B) Representative images from smRNA-FISH analysis of cen(dg) RNA in ago1∆ and ago1∆ cpn1∆ cells. In both A and B, data are representative of at least three independent experiments. (C) Quantification of the mean signal intensity for nuclear cen(dg) RNA foci in ago1∆ cpn1∆ relative to ago1∆ cells, from the smRNA-FISH analysis shown in B. (D) RT-qPCR analysis of total cellular levels of cen(dg) RNA relative to act1+, in ago1∆ cpn1∆ relative to ago1∆ cells. RT-qPCR data are averages of three biological replicates; dots represent individual data points. In all cases, error bars represent one SD, and asterisks denote p ≤ 0.05 (*), or p ≤ 0.001 (***), from Student’s t-test analysis. (TIF) [file pgen.1011620.s008.tif]

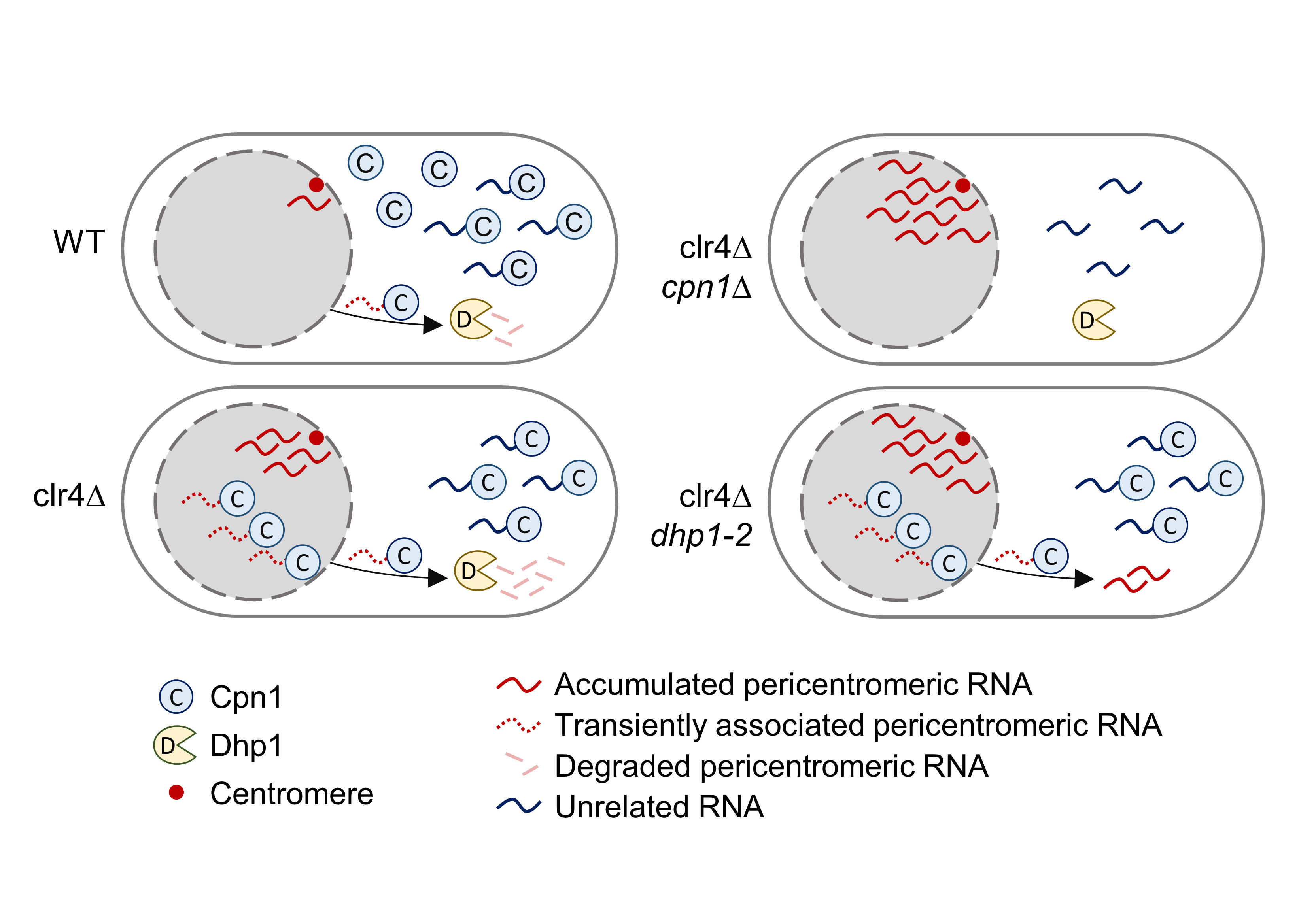

Supplement: S9 Fig — Cpn1 associates with pericentromeric RNAs to target them for Dhp1-dependent degradation. In comparison to wild-type cells, in clr4∆ cells there is increased pericentromeric transcript accumulation, leading to greater Cpn1 association; this reduces the free Cpn1 pool available for stress granule formation in response to stress. Absence of Cpn1 leads to reduced targeting for Dhp1-mediated degradation, and hence hyperaccumulation of pericentromeric transcripts in the nucleus. Absence of Dhp1 results in some accumulation of pericentromeric transcripts in the cytoplasm, suggesting that Cpn1 may help to promote their export for cytoplasmic degradation. (TIF) [file pgen.1011620.s009.tif]
