# Supplementary material for: Fission yeast Caprin protein is required for efficient heterochromatin establishment
Source: PLoS Genet. 2025 Mar 10;21(3):e1011620. doi: 10.1371/journal.pgen.1011620 (PMC11918387; doi:10.1371/journal.pgen.1011620)
Supplement: S1 Table — (DOCX) [file pgen.1011620.s010.docx]

**Table S1. Transcripts upregulated in *cpn1∆* cells (log2FC > 1; FDR < 0.05).**

| **Gene ID** | **Name** | **Description** | **logFC** | **Chr** |
| --- | --- | --- | --- | --- |
| SPBC3E7.02c | hsp16 | heat shock protein Hsp16 | 1.93 | II |
| SPNCRNA.774 |  | non-coding RNA | 1.51 | I |
| SPNCRNA.232 |  | centromeric lncRNA | 1.36 | I |
| SPNCRNA.362 |  | centromeric lncRNA | 1.26 | II |
| SPAC12G12.08 | mrpl1602 | mitochondrial ribosomal protein subunit L6 | 1.24 | I |
| SPNCRNA.230 |  | centromeric lncRNA | 1.23 | I |
| SPNCRNA.1271 |  | non-coding RNA | 1.16 | III |
| SPRRNA.05 |  | 5S rRNA | 1.14 | III |
| SPAC15F9.01c | glm1 | Glomulin, ubiquitin-protein transferase inhibitor | 1.22 | I |
| SPCC663.06c | osr1 | short chain dehydrogenase, unknown specificity | 1.08 | III |
| SPNCRNA.1241 |  | non-coding RNA | 1.01 | III |
|  |  |  |  |  |
