# Supplementary material for: Fission yeast Caprin protein is required for efficient heterochromatin establishment
Source: PLoS Genet. 2025 Mar 10;21(3):e1011620. doi: 10.1371/journal.pgen.1011620 (PMC11918387; doi:10.1371/journal.pgen.1011620)
Supplement: S2 Table — (DOCX) [file pgen.1011620.s011.docx]

**Table S2. Transcripts downregulated in *cpn1∆* cells (log2FC < -1; FDR < 0.05).**

| **Gene ID** | **Name** | **Description** | **logFC** | **Chr** |
| --- | --- | --- | --- | --- |
| SPAC12G12.07c | cpn1 | Caprin | -5.60 | I |
| SPNCRNA.388 |  | non-coding RNA | -3.41 | II |
| SPBPB2B2.06c |  | extracellular 5'-nucleotidase, human NT5E family | -2.83 | II (T) |
| SPBC23G7.10c |  | NADH-dependent flavin oxidoreductase | -2.56 | II |
| SPAC212.08C |  | *S. pombe* specific GPI anchored protein | -2.11 | I (T) |
| SPBCPT2R1.06c |  | pseudogene | -1.82 | II (T) |
| SPAC750.07c |  | *S. pombe* specific GPI anchored protein | -1.79 | I (T) |
| SPAC212.12 |  | *S. pombe* specific GPI anchored protein | -1.60 | I (T) |
| SPBP4G3.02 | pho1 | extracellular acid phosphatase Pho1 | -1.54 | I (T) |
| SPBPB21E7.01c | eno102 | enolase | -1.37 | II (T) |
| SPBPB21E7.07 | aes1 | phenazine biosynthesis PhzF protein family | -1.21 | II (T) |
| SPBC8E4.12c | ecl3 | extender of chronological lifespan protein Ecl3 | -1.18 | II (T) |
| SPBPB8B6.04c | grt1 | DNA-binding transcription factor Grt1 | -1.15 | II (T) |
| SPBC460.04c |  | sulfonate/alpha-ketoglutarate dioxygenase | -1.12 | II (T) |
| SPNCRNA.1487 |  | non-coding RNA | -1.08 | II |
| SPBPB10D8.03 |  | pseudogene transporter | -1.03 | II (T) |

(T) denotes subtelomeric, defined as within 100kb of chromosome end
