# Supplementary material for: Fission yeast Caprin protein is required for efficient heterochromatin establishment
Source: PLoS Genet. 2025 Mar 10;21(3):e1011620. doi: 10.1371/journal.pgen.1011620 (PMC11918387; doi:10.1371/journal.pgen.1011620)
Supplement: S3 Table — (DOCX) [file pgen.1011620.s012.docx]

**S3 Table. Yeast strains**

| **Strain** | **Genotype** | **Figure** |
| --- | --- | --- |
| 7 | *h+ otr1R:ade6+ ade6-210 leu1-32 ura4-D18* | 1,2,3,S2,S4,S5,S6 |
| 131 | *h- dcr1::NatMX6 ade6-210 leu1-32 ura4-D18 arg3-D4 his3D1* | 3 |
| 140 | *h- sir2::NatMX6 ade6-210 otrR:ade6+ ura4-D18* | S3 |
| 1621 | *h+ rrp6::KanMX6 ade6-210 ura4-D18 leu1-32* | 2,S2 |
| 1952 | *h- ago1::ura4+ otr1R:ade6+ ade6-210 ura4-D18 leu1-32* | 2 |
| 2233 | *rrp6::KanMX6 mkt1::NatMX6 otr1R:ade6+ ade6-210 leu1-32 ura4-D18* | 2,S2 |
| 2505 | *rrp6::KanMX6 dcr1::NatMX6 otr1R:ade6+ ade6-210 leu1-32 ura4-DSE* | 2,S2 |
| 4471 | *h90 clr4::leu2+ cc2:his3+ ade6-704-NatMX6 ura4-DSE/D18 leu1-32 his3-D1* | 2 |
| 4472 | *h+ cc2:his3+ ade6-704-HygMX6 ura4-DSE/D18 leu1-32 his3-D1 arg3-D4* | 2 |
| 4512 | *h- sir2::ura4+ Mkt1-Flag-NatMX6 otr1R:ade6+ ade6-210 leu1-32 ura4-D18* | S3 |
| 4578 | *h- rik1::HygMX6:ura4+ otr1R:ade6+ ade6-210 leu1-32 ura4-D18 his3-D1* | 1,2,3,7,S1,S5 |
| 4658 | *h+ dcr1::NatMX6:ura4+ otr1R:ade6+ ade6-210 leu1-32 ura4-D18* | S1 |
| 5135 | *h+ ago1::NatMX6:ura4+ otr1R:ade6+ ade6-210 leu1-32 ura4-D18* | 1,2,3,7,S1,S5 |
| 5288 | *h+ stc1::NatMX6:ura4+ otr1R:ade6+ ade6-210 leu1-32 ura4-D18* | S1 |
| 6407 | *red1::KanMX6 otr1R:ade6+ ade6-210 leu1-32 ura4-D18 arg3-D4* | S2 |
| 6601 | *h90 ago1::NatMX6:ura4+ lys1::leu1+ otr1R:ade6+ ade6-210 leu1-32 ura4-D18* | 1,S1 |
| 6605 | *h- rik1::HygMX6:ura4+ lys1::leu1+ otr1R:ade6+ ade6-210 leu1-32 ura4-D18* | 1,S1 |
| 6633 | *h+ rik1::HygMX6:ura4+ ago1::NatMX6:ura4+ lys1::leu1+ otr1R:ade6+ ade6-210 leu1-32 ura4-D18* | 1 |
| 6639 | *h- ago1::NatMX6:ura4+ otr1R:ade6+ ade6-DN/N leu1-32 ura4-D18 his3-D1* | S1 |
| 6751 | *h- ago1::NatMX6:ura4+ mkt1::KanMX6 otr1R:ade6+ ade6-210 leu1-32 ura4-D18* | 1 |
| 6759 | *h+ ago1::NatMX6:ura4+ tri1::KanMX6 otr1R:ade6+ ade6-210 leu1-32 ura4-D18* | 1 |
| 6765 | *h+ rik1::HygMX6:ura4+ mkt1::KanMX6 otr1R:ade6+ ade6-210 leu1-32 ura4-D18* | 1 |
| 6798 | *h- rik1::HygMX6:ura4+ tri1::KanMX6 otr1R:ade6+ ade6-210 leu1-32 ura4-D18* | 1 |
| 6928 | *h+ otr1R:ade6+ lys1::leu1+ ura4+ ade6-210 leu1-32* | 1 |
| 6974 | *h- clr4::HygMX6:ura4+ otr1R:ade6+ ade6-210 leu1-32 ura4-D18 his3-D1* | 1,2,3,6,7,S1 |
| 7089 | *rik1::HygMX6:ura4+ otr1R:ade6+ ade6-DN/N leu1-32 ura4-D18 his3-D1* | S1 |
| 7120 | *h+ PSP102-Sir2 leu2+ rik1::HygMX6:ura4+ otr1R:ade6+ ade6-210 leu1-32 ura4-D18 his3-D1* | 1 |
| 7123 | *h- PSP102-Sir2 leu2+ ago1::NatMX6:ura4+ otr1R:ade6+ ade6-210 leu1-32 ura4-D18 his3-D1* | 1 |
| 7124 | *h+ PSP102-Clr3 leu2+ rik1::HygMX6:ura4+ otr1R:ade6+ ade6-210 leu1-32 ura4-D18 his3-D1* | 1 |
| 7129 | *h- PSP102-Clr3 leu2+ ago1::NatMX6:ura4+ otr1R:ade6+ ade6-210 leu1-32 ura4-D18 his3-D1* | 1 |
| 7130 | *h+ PSP102-Sir2&Clr3 leu2+ rik1::HygMX6:ura4+ otr1R:ade6+ ade6-210 leu1-32 ura4-D18 his3-D1* | 1 |
| 7133 | *h- PSP102-Sir2&Clr3 leu2+ ago1::NatMX6:ura4+ otr1R:ade6+ ade6-210 leu1-32 ura4-D18 his3-D1* | 1 |
| 7221 | *h- gcn5::KanMX6 rik1::HygMX6:ura4+ otr1R:ade6+ ade6-210 leu1-32 ura4-D18* | 1 |
| 7225 | *h+ gcn5::KanMX6 ago1::NatMX6:ura4+ otr1R:ade6+ ade6-210 leu1-32 ura4-D18* | 1 |
| 7577 | *h+ cpn1::HygMX6 otr1R:ade6+ ade6-210 leu1-32 ura4-D18* | 2,3,S2,S4 |
| 7669 | *h+ cpn1::KanMX6 rik1::HygMX6:ura4+ otr1R:ade6+ ade6-210 leu1-32 ura4-D18 his3-D1* | 2,3 |
| 7706 | *h- cpn1::KanMX6 ago1::NatMX6:ura4+ otr1R:ade6+ ade6-210 leu1-32 ura4-D18* | 2,3 |
| 7707 | *h+ cpn1::KanMX6 ago1::NatMX6:ura4+ otr1R:ade6+ ade6-210 leu1-32 ura4-D18* | 2 |
| 7805 | *h- ago1::ura4+ otr1R:ade6+ lys1::NatMX6 ade6-210-HygMX6 mat1_m-cyhS smt0 CycR leu1-32 ura4-D18* | S1 |
| 7905 | *h+ rik1::ura4+ otr1R:ade6+ lys1::NatMX6 ade6-210-HygMX6 ura4-D18 leu1-32* | S1 |
| 8457 | *rrp6::KanMX6 cpn1::Hyg otr1R:ade6+ ade6-210 ura4-D18 leu1-32* | 2,S2 |
| 8543 | *h+ Cpn1-GFP-KanMX6 otr1R::ade6+ ade6-210 leu1-32 ura4-D18* | 6,S5,S6 |
| 8603 | *h+ cpn1^R6A^ otr1R:ade6+ ade6-210 leu1-32 ura4-D18* | 2,3 |
| 8650 | *rrp6::KanMX6 tri1::leu1+ otr1R:ade6+ ade6-210 ura4-D18 leu1-32* | 2,S2 |
| 8725 | *h+ cpn1^R6A^ ago1::NatMX6:ura4+ otr1R:ade6+ ade6-210 leu1-32 ura4-D18* | 2 |
| 8776 | *rrp6::KanMX6 cpn1^R6A^ otr1R:ade6+ ade6-210 ura4-D18 leu1-32* | 2,S2 |
| 8800 | *cpn1::KanMX6 cc2::his3+ ade6-704-HygMX6 ura4-DSE/D18 leu1-32* | 2 |
| 8314 | *h+ Cpn1-Flag-NatMX6 otr1R:ade6+ ade6-210 leu1-32 ura4-D18* | 3 |
| 8681 | *h- cpn1^R6A^ rik1::HygMX6:ura4+ otr1R:ade6+ ade6-210 leu1-32 ura4-D18* | 3 |
| 8725 | *h+ cpn1^R6A^ ago1::NatMX6:ura4+ otr1R:ade6+ ade6-210 leu1-32 ura4-D18* | 3 |
| 8785 | *rik1::HygMX6:ura4+ Cpn1-GFP-KanMX6 otr1R:ade6+ ade6-210 leu1-32 ura4-D18* | S5,S6 |
| 8787 | *ago1::NatMX6:ura4+ Cpn1-GFP-KanMX6 otr1R:ade6+ ade6-210 leu1-32 ura4-D18* | S5.S6 |
| 8789 | *h+ Nxt3-GFP-KanMX6 otr1R:ade6+ ade6-210 leu1-32 ura4-D18* | S5 |
| 8798 | *cpn1::NatMX6 clr4::HygMX6:ura4+ otr1R:ade6+ ade6-210 leu1-32 ura4-D18* | 7 |
| 8804 | *h+ Ubp3-GFP-KanMX6 otr1R:ade6+ ade6-210 leu1-32 ura4-D18* | S5 |
| 8834 | *h+ Cpn1^R6A^-GFP-KanMX6 otr1R:ade6+ ade6-210 leu1-32 ura4-D18* | S5 |
| 8835 | *h+ ubp3::HA-KanMX6 otr1R:ade6+ ade6-210 leu1-32 ura4-D18* | 3 |
| 8854 | *h+ Cpn1^R6A^-Flag-NatMX6 otr1R:ade6+ ade6-210 leu1-32 ura4-D18* | 3 |
| 8869 | *clr4::HygMX6:ura4+ Cpn1-GFP-KanMX6 otr1R:ade6+ ade6-210 leu1-32 ura4-D18* | 6,S6 |
| 8872 | *Nxt3-GFP-KanMX6 rik1::HygMX6:ura4+ otr1R:ade6+ ade6-210 leu1-32 ura4-D18 his3-D1* | S5 |
| 8874 | *Ubp3-GFP-KanMX6 rik1::HygMX6:ura4+ otr1R:ade6+ ade6-210 leu1-32 ura4-D18 his3-D1* | S5 |
| 8878 | *Nxt3-GFP-KanMX6 ago1::NatMX6:ura4+ otr1R:ade6+ ade6-210 leu1-32 ura4-D18* | S5 |
| 8880 | *Ubp3-GFP-KanMX6 ago1::NatMX6:ura4+ otr1R:ade6+ ade6-210 leu1-32 ura4-D18* | S5 |
| 8928 | *ubp3::HA-KanMX6 rik1::HygMX6:ura4+ otr1R::ade6+ ade6-210 leu1-32 ura4-D18* | 3 |
| 8930 | *ubp3::HA-KanMX6 ago1::NatMX6:ura4+ otr1R::ade6+ ade6-210 leu1-32 ura4-D18* | 3 |
| 8948 | *h- nxt3::HA-KanMX6 otr1R:ade6+ ade6-210 leu1-32 ura4-D18* | 3 |
| 9031 | *nxt3::HA-KanMX6 rik1::HygMX6:ura4+ otr1R:ade6+ ade6-210 leu1-32 ura4-D18* | 3 |
| 9033 | *nxt3::HA-KanMX6 ago1::NatMX6:ura4+ otr1R:ade6+ ade6-210 leu1-32 ura4-D18* | 3 |
| 9144 | *h+ Cpn1-GFP-KanMX6 Pabp-mRFP-HygMX6 leu1-32 ura4-D18* | 4,5,S7 |
| 9145 | *h+ Cpn1^R6A^-GFP-KanMX6 Pabp-mRFP-HygMX6 leu1-32 ura4-D18* | 4 |
| 9147 | *h+ cpn1::NatMX6 Pabp-mRFP-HygMX6 leu1-32 ura4-D18* | 4,5 |
| 9151 | *h+ Nxt3-GFP-KanMX6 Pabp-mRFP-HygMX6 leu1-32 ura4-D18* | 4 |
| 9152 | *h+ Ubp3-GFP-KanMX6 Pabp-mRFP-HygMX6 leu1-32 ura4-D18* | 4 |
| 9225 | *mkt1::NatMX6 Cpn1-GFP-KanMX6 Pabp-mRFP-HygMX6 leu1-32 ura4-D18* | 4,5 |
| 9226 | *ubp3::NatMX6 Cpn1-GFP-KanMX6 Pabp-mRFP-HygMX6 leu1-32 ura4-D18* | 4 |
| 9232 | *cpn1^R6A^ Pabp-mRFP-HygMX6 leu1-32 ura4-D18* | 4 |
| 9263 | *clr4::NatMX6 Cpn1-GFP-KanMX6 Pabp-mRFP-HygMX6 leu1-32 ura4-D18* | 5,S7 |
| 9297 | *nxt3::NatMX6 Cpn1-GFP-KanMX6 Pabp-mRFP-HygMX6 leu1-32 ura4-D18* | 4 |
| 9306 | *h90 ago1::ura4+ Cpn1-GFP-KanMX6 Pabp-mRFP-HygMX6 leu1-32 ura4-D18* | 5,S7 |
| 9327 | *Cpn1-mCherry-NatMX6 Nxt3-GFP-KanMX6 otr1R:ade6+ ade6-210 leu1-32 ura4-D18* | 4 |
| 9329 | *Cpn1-mCherry-NatMX6 Ubp3-GFP-KanMX6 otr1R:ade6+ ade6-210 leu1-32 ura4-D18* | 4 |
| 9413 | *sir2::ura4+ Cpn1-Flag-NatMX6 ade6-210 leu1-32 ura4D18* | S3 |
| 9418 | *sir2::ura4+ cpn1::KanMX6 Mkt1-Flag-NatMX6 ade6-210 leu1-32 ura4-D18* | S3 |
| 9440 | *rik1::ura4+ Cpn1-GFP-KanMX6 Pabp-mRFP-HygMX6 leu1-32 ura4-D18* | 5,S7 |
| 9520 | *h- ago1::ura4+ Pabp-GFP-KanMX6 otr1R:ade6+ ade6-210 leu1-32 ura4-D18* | 5,S8 |
| 9527 | *clr4::HygMX6:ura4+ Cpn1-GFP-KanMX6 otr1R:ade6+ ade6-210 leu1-32 ura4-D18* | 6 |
| 9529 | *h- clr4::HygMX6:ura4+ Pabp-GFP-KanMX6 otr1R:ade6+ ade6-210 leu1-32 ura4-D18* | 5,6,7 |
| 9536 | *clr4::HygMX6 GFP-Cnp1-NatMX6 leu1-32 ura4-D18* | 6 |
| 9538 | *ago1::ura4+ cpn1::NatMX6 Pabp-GFP-KanMX6 ade6-210 ura4-D18 leu1-32* | 5,S8 |
| 9542 | *clr4::HygMX6:ura4+ cpn1::NatMX6 Pabp-GFP-KanMX6 ade6-210 leu1-32 ura4-D18* | 5,7 |
| 9586 | *clr4::HygMX6:ura4+ dhp1-2 (NatMX6) Pabp-GFP-KanMX6 otr1R:ade6+ ade6-210 leu1-32 ura4-D18* | 7 |
| 9596 | *clr4::HygMX6:ura4+ dhp1-2 (NatMX6) otr1R:ade6+ ade6-210 leu1-32 ura4-D18* | 7 |
| 9609 | *clr4::HygMX6:ura4+ cpn1::KanMX6 dhp1-2 (NatMX6) otr1R:ade6+ ade6-210 leu1-32 ura4-D18* | 7 |
| 9636 | *rik1::ura4+ dhp1-2 (NatMX6) otr1R:ade6+ ade6-210-HygMX6 leu1-32 ura4-D18* | 7 |
| 9638 | *ago1::ura4+ dhp1-2 (NatMX6) otr1R:ade6+ ade6-210-HygMX6 leu1-32 ura4-D18* | 7 |
| 9658 | *clr4::HygMX6:ura4+ dhp1-2 (NatMX6) Cpn1-GFP-KanMX6 otr1R:ade6+ ade6-210? leu1-32 ura4-D18/DSE* | 7 |
|  |  |  |
