# Supplementary material for: Fission yeast Caprin protein is required for efficient heterochromatin establishment
Source: PLoS Genet. 2025 Mar 10;21(3):e1011620. doi: 10.1371/journal.pgen.1011620 (PMC11918387; doi:10.1371/journal.pgen.1011620)
Supplement: S4 Table — (DOCX) [file pgen.1011620.s013.docx]

**S4 Table. qPCR primers**

| Name | Sequence |
| --- | --- |
| q_act1_F | GGTTTCGCTGGAGATGATG |
| q_act1_R | ATACCACGCTTGCTTTGAG |
| q_dg_F | AATTGTGGTGGTGTGGTAATAC |
| q_dg_R | GGGTTCATCGTTTCCATTCAG |
| q_imr_F | CTAATGCGGAGTAAGGCTAATC |
| q_imr_R | TGGACAGAATGGATGGATATTG |
| q_mei4_F | AAAAGCGACCTTCAAGCAAA |
| q_mei4_R | TTGCATCGTTTGAGACTTCG |
| q_ssm4_F | AACAGCTAAAGACCGCAAGG |
| q_ssm4_R | TCTCCTTGCAGGCAAAGGTC |
| q_RPT6_207F | GAGAATCCATTCGAGGTCCA |
| q_RPT6_207R | AATGCAAACATACCGGCTTC |
| q_SPCC1442.04_110F | CCAAACTCTGTTGTTGCAGAAG |
| q_SPCC1442.04_110R | GATTCCTCAAGGTCGTTATCCC |
| q_Tf2-5_F | AGGGCTGTAAGACAATAGTGAAG |
| q_Tf2-5_R | AGGTCGGTAGTCGATATACCAT |
| q_Tf2-3_F | GTTCACAAATCGGTTTCCAG |
| q_Tf2-3_R | GCGTGCACTATAGTGAACAATC |
| q_MC-dgR_F | ATCATTCAGAAAATCACCGGAGCAAT |
| q_MC-dgR_R | TCGCCCTAAAAGTAAACGGTAAGC |
