# Supplementary material for: Fission yeast Caprin protein is required for efficient heterochromatin establishment
Source: PLoS Genet. 2025 Mar 10;21(3):e1011620. doi: 10.1371/journal.pgen.1011620 (PMC11918387; doi:10.1371/journal.pgen.1011620)
Supplement: S5 Table — (DOCX) [file pgen.1011620.s014.docx]

**S5 Table. smRNA FISH probes**

| TTTCCTATTGCAATGAATGTGA |
| --- |
| CCGATTGTATATACCGAATACA |
| TATACTTGGTACAACATGCAAT |
| ACTTCAAAATCTCATCACTATA |
| TTCCATGTGGTAATAGAACACA |
| ACAACGATTATGGTCTCCACTC |
| GGATAAAGTTTGTCAACATACT |
| GAAGAACTGAGTTTTTACCCTT |
| CAACTCTGGTCCGATTTATAAC |
| CTGTTCACTTATCTAATTCGTT |
| ATACTGGTTTGTACTTGCTAAG |
| TTACCTTTGTTAATTCAATTCT |
| CAATGAGTAAACTCCACTAAGA |
| ACCATTCAACTTGGCAAGTATA |
| GATACTGGTGATGATGTTGATG |
| AGAATGGAGATGAAAGTGGTGA |
| CGTACAACAAGCCAATAATGAC |
| GTACATACGTTGAAACATTTGA |
| GCTAACAAGAAATCGTAGTCAC |
| TCTTTATCCTCTTCGTTTATAT |
| CGGCCAAAATTATTTACTCCAA |
| TTATAGTGTGAAGCCACTTTCC |
| AATGTATTAGCTGTGTCAACCA |
| GAATGCTGAGAAAGTTTATGTC |
| CCAATAGTATTTTTACTGTTCG |
| ACTATATATCCATGAATCATTT |
| ATACGATATCGGTTCAATCACA |
| ATGATGTAGAAGATGTACAGGA |
| ATTTATGATTCTTCCACATGTT |
| CGATTGAGACATTATTTTTGTG |
| AGTCAGAGCAGACAATACGTAT |
| GCATATTGGTCGTTTTTAATT |
| CATGATGTTAAGGGTGAACCG |
| TATTTTACTACCCATGATGTC |
| TTTAATATCTGGTGTTGTGGA |
| CTGCGGATGGAAAAAGTTCTT |
| AAGTAGGAATGATGTACTCCC |
| GCCATAAATATATGACCTAGA |
| GCATAGCGATGATAGTTCTAA |
| GCTTTTAGTGCGGTCATTTAA |
| TCTTTCTATGTTCGAGCTTTG |
| ATCTTTAAATCCCTTTCTGGA |
| ACTCGCATCTTTATATCTGAT |
| TTTGTGCGGAATGTCTACTTC |
| AGCACAAGAGACATGGTGTAC |
| ATGATAACGGATCTAGCTTCG |
| TCCTGGATATCCTTTGTAATA |
| ACATATCGGTTTTCATTGTGT |
